# A sleepy cannabis constituent: cannabinol and its active metabolite influence sleep architecture in rats

**DOI:** 10.1038/s41386-024-02018-7

**Published:** 2024-11-12

**Authors:** Jonathon C. Arnold, Cassandra V. Occelli Hanbury-Brown, Lyndsey L. Anderson, Miguel A. Bedoya-Pérez, Michael Udoh, Laura A. Sharman, Joel S. Raymond, Peter T. Doohan, Adam Ametovski, Iain S. McGregor

**Affiliations:** 1 Lambert Initiative for Cannabinoid Therapeutics, The University of Sydney, Sydney, NSW Australia; 2 Discipline of Pharmacology, School of Pharmacy, Faculty of Medicine and Health, The University of Sydney, Sydney, NSW Australia; 3 Brain and Mind Centre, The University of Sydney, Sydney, NSW Australia; 4 School of Psychology, Faculty of Science, The University of Sydney, Sydney, NSW Australia; 5 School of Chemistry, Faculty of Science, The University of Sydney, Sydney, NSW Australia

**Keywords:** Preclinical research, Drug development

## Abstract

Medicinal cannabis is being used worldwide and there is increasing use of novel cannabis products in the community. Cannabis contains the major cannabinoids, Δ^9^-tetrahydrocannabinol (Δ^9^-THC) and cannabidiol (CBD), but also an array of minor cannabinoids that have undergone much less pharmacological characterization. Cannabinol (CBN) is a minor cannabinoid used in the community in “isolate’ products and is claimed to have pro-sleep effects comparable to conventional sleep medications. However, no study has yet examined whether it impacts sleep architecture using objective sleep measures. The effects of CBN on sleep in rats using polysomnography were therefore examined. CBN increased total sleep time, although there was evidence of biphasic effects with initial sleep suppression before a dramatic increase in sleep. CBN increased both non-rapid eye movement (NREM) and rapid eye movement (REM) sleep. The magnitude of the effect of CBN on NREM was comparable to the sleep aid zolpidem, although, unlike CBN, zolpidem did not influence REM sleep. Following CBN dosing, 11-hydroxy-CBN, a primary metabolite of CBN surprisingly attained equivalently high brain concentrations to CBN. 11-hydroxy-CBN was active at cannabinoid CB_1_ receptors with comparable potency and efficacy to Δ^9^-THC, however, CBN had much lower activity. We then discovered that the metabolite 11-hydroxy-CBN also influenced sleep architecture, albeit with some subtle differences from CBN itself. This study shows CBN affects sleep using objective sleep measures and suggests an active metabolite may contribute to its hypnotic action.

## Introduction

Medicinal cannabis is legal in many countries and there is increasing use of cannabis products. Cannabis contains ~500 compounds of which ~125 are cannabinoids (or more specifically phytocannabinoids) [1]. These include the major cannabinoids: Δ^9^-tetrahydrocannabinol (Δ^9^-THC), the main psychoactive constituent of cannabis, and cannabidiol (CBD), a non-intoxicating constituent [2, 3]. Community surveys of medicinal cannabis use reveal increasing numbers of patients who utilize cannabis-based products for sleep problems [4, 5]. Sleep disorders are highly prevalent, with estimates that up to half of the population report insomnia symptoms, with one-fifth having an insomnia disorder [6–8]. Current pharmaceutical sleep therapies have issues with efficacy but also side-effect liabilities, which helps explain why many individuals seek out alternative therapies like cannabis products.

While the major phytocannabinoids Δ^9^-THC and CBD have undergone extensive pharmacological characterization, little is known about the pharmacology and therapeutic potential of the “minor” phytocannabinoids [9–13]. There is an increasing use of highly purified minor cannabinoid “isolate” products, especially in the United States, but this occurs in the absence of objective scientific evidence. One particular minor phytocannabinoid being used in such products is cannabinol (CBN), which was the first cannabinoid discovered in 1896 [14, 15]. This molecule is an oxidative metabolite of Δ^9^-THC and thus accumulates in aged cannabis due to exposure to air, heat, and light. Cannabis folklore holds that aged cannabis has remarkable soporific effects and that CBN is the responsible active constituent [16, 17]. CBN is often referred to as the ‘sleepy cannabinoid’, with claims of profound pro-sleep effects matching that of conventional sleep drugs such as benzodiazepines. Indeed, some manufacturers of CBN isolate products market it as a sleep aid. However, there is scant objective scientific evidence to support CBN’s use as a sedative, hypnotic agent.

Endocannabinoids such as anandamide and 2-arachidonoyl glycerol (2-AG) target the cannabinoid CB_1_ receptor, which is responsible for the intoxicating and sedative effects of Δ^9^-THC [3]. CBN is known to bind and activate CB_1_ receptors, although with much lower potency than Δ^9^-THC [18]. CBN may then impact sleep via its effects on the CB_1_ receptor as part of the endogenous cannabinoid system, which is known to regulate sleep-wake cycles [19]. Indeed, genetic deletion or pharmacological blockade of the CB_1_ receptor disrupts both non-rapid eye movement (NREM) and rapid eye movement (REM) sleep, while CB_1_ receptor activation increases NREM sleep [20, 21]. However, there is contention around the CB_1_ receptor pharmacology of CBN, with some studies showing it is inactive at human CB_1_ receptors [22, 23]. Consistent with this, most studies that administered CBN to humans reported no Δ^9^-THC-like intoxicating effects or somnolence [16, 24].

Those claiming that CBN improves sleep often refer to a study conducted almost 50 years ago which showed CBN prolonged pentobarbital-induced sleep time in rats [25]. Although, more recently two human studies administered CBN to healthy participants and reported promising sleep improvements using subjective measures of sleep quality [26, 27]. However, no study has hitherto examined whether CBN impacts more objective measures of sleep using polysomnography, which measures sleep-related electrical activity in the cortex to elucidate sleep stages such as NREM and REM sleep. Here we used a wireless polysomnographic telemetry system in rats to assess the effects of CBN on various measures of sleep architecture and compared the effects to a common sleep aid, zolpidem. We explored mechanisms by performing a pharmacokinetic study to observe brain and plasma concentrations of CBN and its major metabolites, and the action of these compounds on cannabinoid receptors. We then assessed the effects of a potential active metabolite of CBN on sleep.

## Materials and methods

Below is a condensed version of the Materials and Methods. Detailed Materials and Methods can be found in the Supplementary Materials.

### Rats

A total of 62 male 9-week-old Long-Evans rats were sourced from the Animal Resources Centre (Murdoch, Western Australia). Animals were randomly assigned to polycarbonate cages and housed in groups of three. When recovering from surgery, during telemetry recording or habituation to recording, rats were housed singly in polycarbonate cages situated above telemetry plates. All cages had standard bedding and enrichment and rats had unrestricted access to food and water. Holding cages were maintained in a climate-controlled room (humidity 30–70%: ambient temperature 22 °C) under an artificial 12:12 h light cycle where lights on were considered the zeitgeber (ZT) and ZT0 signified lights on and ZT12 lights off. The transition between light phases occurred as a 15 min gradual fade between 500 and 0 lux. The experimental protocols for the study were approved by the University of Sydney Animal Ethics Committee and were in accordance with the Australian Code of Practice for the Care and Use of Animals for Scientific Purposes.

### Drugs

Highly purified CBN was sourced from THCPharm (Germany) (purity 100%); see Fig. S6 for in-house purity analysis with LC–MS/MS. Zolpidem was sourced from Sigma–Aldrich (St Louis, USA). Samples of 11-OH-CBN and 11-COOH-CBN for pharmacokinetic analysis were synthesized in-house by Dr Adam Ametovski. All formulations were prepared fresh and dissolved in a vehicle composed of 1:1:18 parts ethanol, Tween 80, and 0.9% physiological saline administered intraperitoneally (IP) at 5 ml/kg. As they were insoluble at the required volumes, doses of 30 mg/kg and 100 mg/kg of CBN were delivered as suspensions.

### Drug administration and polysomnographic recordings

After a two-week recovery from surgery (see details in the Supplementary Materials and Methods), three baseline recordings were made in which rats were habituated to receiving IP saline injections and the treatment regimen began the following week. Our studies used HD-X02 biotelemetry probes from Data Sciences International (DSI, Minneapolis, USA), which allowed the measurement of EEG, EMG, subcutaneous body temperature, and locomotor activity. On recording days, rats were weighed and placed in telemetry cages at ZT11 allowing them to habituate to their new environment before recording. EEG and EMG were collected from ZT12 up to ZT0. It is common practice in hypnotic drug discovery to administer drugs in the dark phase when rodents are relatively active. This is because (1) rodents exhibit polyphasic sleep, so NREM and REM sleep can be measured in this period, although sleep pressure is diminished; (2) it better mirrors insomnia patients who have reduced sleep pressure; and (3) it helps avoid false negatives due to ceiling effects conferred by testing in the light/inactive phase [28, 29]. At ZT13, all rats were injected with respective treatments by an experimenter blind to treatment conditions and returned immediately to their respective cages within 15 minutes. Once the recording session was completed, the animals were returned to group housing.

For the acute dose-response study to CBN or 11-OH-CBN, eight rats received injections of vehicle, 10, 30, or 100 mg/kg CBN or vehicle, 1, 3, and 10 mg/kg 11-OH-CBN as per a randomized Latin square design. Each rat received each treatment condition once, separated by a standard 5-day washout period to avoid potential tolerance and/or residual CBN or metabolites being present at the time of testing.

To provide a positive control and a point of comparison for the effects of acute CBN on sleep architecture, we performed a follow-up experiment in the same rats using zolpidem which induces robust hypnotic effects in rats [30, 31]. Zolpidem 10 mg/kg IP and vehicle were administered over two treatment sessions as per a randomized cross-over design conforming to the same protocol as the initial experiment.

In the repeated dosing experiment, eight rats were randomly assigned to vehicle condition and eight to the CBN 10 mg/kg condition. Rats were administered the same treatment at ZT13 each day for 15 days, however, telemetry recordings were taken on days 1, 8, and 15 of respective treatments, and the response was compared between subjects.

Polysomnographic data were recorded using DSI Ponemah software (Version 6.41, Minneapolis, USA) [32] from freely moving rats which was transmitted via the biotelemetry probes to receiver plates that were connected to a matrix 2 (MX-2) and relayed to a computer located in an adjacent room. DSI NeuroScore software (version 3.2.1) (Minneapolis, USA) was used to visualize the data in 10 s epochs to enable manual scoring of the bouts, duration of bouts, and latency times of the various sleep parameters (active wake, quiet wake, NREM sleep, REM sleep, NREM and REM sleep onset latencies, and total sleep time (TST)). This was performed by a single experimenter blind to experimental conditions. For details of the analysis and scoring criteria of the various sleep parameters see the Supplementary Materials and Methods.

### Pharmacokinetic analysis

Rats received 10 mg/kg CBN IP at approximately ZT13. Rats were then anesthetized with isoflurane at selected ZT points (13, 13.5, 14, 15, 17, 19, and 21) and blood was collected by terminal cardiac puncture. Brains were harvested, snap frozen on dry ice and plasma was isolated via centrifugation at 9000 × *g* for 10 min at 4 °C. All tissues were stored at −80 °C until analysis. CBN and metabolite concentrations in plasma and brain samples were assayed by liquid chromatography-tandem mass spectrometry (LC–MS/MS) using a using a Shimadzu Nexera LC-30AD UHPLC system coupled to a Shimadzu SPD-20AV photodiode array detector and a Shimadzu LC–MS-8040 triple-quadrupole mass spectrometer, equipped with an electrospray ionization (ESI) source (Shimadzu Corp.; Kyoto, Japan) as described previously [33].

### In vitro functional characterization at human CB_1_ and CB_2_ receptors

Functional assays were carried out as previously described in mouse AtT20 FlpIn adenocarcinoma cells stably transfected with human CB_1_ or CB_2_ receptors [34].

### Medicinal chemistry

All reactions were performed under an atmosphere of nitrogen unless otherwise specified. Details are provided in the Supplementary Materials.

### Statistical analyses

Statistical analyses were accomplished using Generalized Linear Mixed Models (GLMM) using R v.4.2.1 [35]. P-values were generated by the type III Wald chi-square test using the function *Anova* from the package “car” [36]. Planned pairwise comparisons were performed using Dunn-Šidák corrections through the function *emmeans* from the package “emmeans” [37]. Statistical significance was defined by α = 0.05.

## Results

### Acute administration of CBN increased sleep in rats

**Fig. 1:**
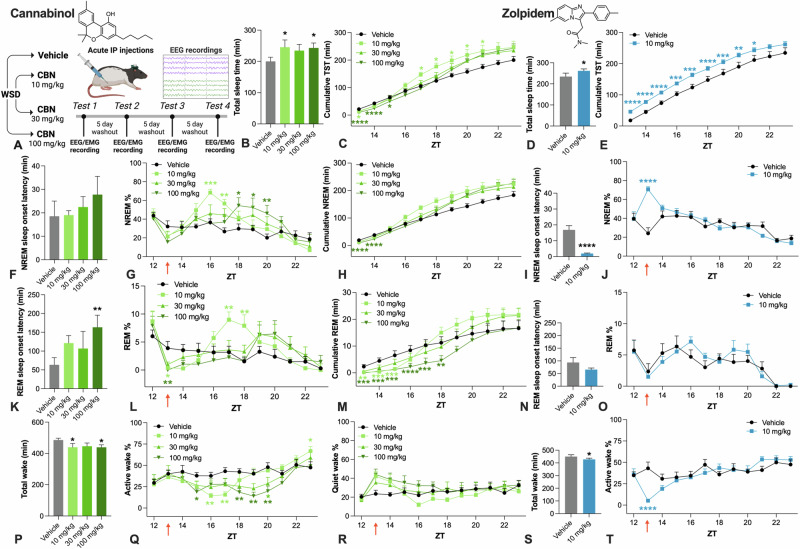
Effects of acute CBN and zolpidem on sleep and wake in rats. **A** Study design. **B** CBN increased total sleep time (χ^2^_3_ = 10.732, *P* = 0.013), but had biphasic effects on **C** cumulative sleep time (CBN main effect, χ^2^_3_ = 19.908, *P* = 0.013; CBN and time interaction, χ^2^_30_ = 85.65, *P* < 0.0001). Zolpidem increased **D** total sleep time and **E** cumulative total sleep time (CBN treatment effects, χ^2^_1_ = 6.057, *P* = 0.014; χ^2^_1_ = 62.428, *P* < 0.0001 respectively). CBN did not affect **F** NREM sleep onset latency but increased **G** % NREM sleep (CBN and time interaction, χ^2^_30_ = 74.4289, *P* < 0.0001_)_, and had a biphasic effect on **H** cumulative NREM time (CBN treatment, χ^2^_3_ = 66.926, *P* < 0.0001; CBN and time interaction, χ^2^_30_ = 73.541, *P* < 0.0001). Zolpidem decreased **I** NREM sleep onset latency and increased **J** % NREM (zolpidem main effect, χ^2^_1_ = 72.941, *P* < 0.0001; zolpidem by time interaction, χ^2^_10_ = 83.479, *P* < 0.0001). CBN increased **K** REM sleep onset latency (main effect CBN, χ^2^_3_ = 14.172, *P* = 0.003). CBN initially suppressed **L** % REM sleep, which was followed by a transient increase in % REM at 10 mg/kg only (main effect of CBN, χ^2^_3_ = 13.655, *P* = 0.003; CBN and time interaction, χ^2^_30_ = 76.089, *P* < 0.0001). CBN similarly influenced **M** cumulative REM time (CBN treatment, χ^2^_3_ = 17.332, *P* < 0.0001; CBN and time interaction, χ^2^_30_ = 91.312, *P* < 0.0001). Zolpidem did not affect **N** REM sleep onset latency, or **O** % REM sleep. CBN decreased **P** total wake time and decreased **Q** % active wake (CBN and time interaction, χ^2^_30_ = 62.048, *P* = 0.001), and affected **R** % quiet wake (CBN main effect; χ^2^_3_ = 10.340, *P* = 0.016). Zolpidem decreased **S** total wake time and decreased **T** % active wake (zolpidem main effect, χ^2^_1_ = 105.464, *P* < 0.0001, zolpidem by time interaction, χ^2^_10_ = 117.882, *P* < 0.0001). Time is expressed relative to lights on (ZT). Dunn–Šidák corrected multiple comparisons test *P < 0.05, **P < 0.01, ***P < 0.001, ****P < 0.0001. Error bars display ± SEM, *n* = 8 per group. CBN cannabinol, WSD within-subjects design (Latin square), EEG electroencephalography, EMG electromyography, IP intraperitoneal, NREM non-rapid eye movement sleep, REM rapid eye movement sleep, ZT zeitgeber time. Created with BioRender.com.

CBN was administered to rats to observe its effects on sleep architecture (Fig. 1A). Acute administration of CBN or zolpidem increased total sleep time with a similar magnitude of effect (Fig. 1B, D). CBN initially decreased cumulative total sleep time, whereas from ZT17 only the low 10 mg/kg dose significantly increased cumulative total sleep time (Fig. 1C). Zolpidem did not display any such rebound effect as evidenced by cumulative total sleep time (Fig. 1E). CBN did not affect NREM sleep onset latency, unlike zolpidem which rapidly induced sleep (Fig. 1F, I). Both zolpidem and CBN increased % NREM sleep, although zolpidem had an immediate effect, whereas CBN had a delayed onset of action, 3–4 h post-dosing (Fig. 1G, J).

CBN 10 mg/kg and 100 mg/kg significantly increased the % NREM sleep compared to vehicle at various time points from ZT16–20, with a longer duration of action than zolpidem. The magnitude of the effect of CBN 10 mg/kg on % NREM sleep was comparable to zolpidem 10 mg/kg, but the latter had a more immediate effect at ZT13–14. CBN produced longer, uninterrupted NREM sleep bouts as it increased both NREM sleep duration and decreased the number of NREM sleep bouts (Fig. S1). Zolpidem, however, increased NREM sleep duration but the number of NREM sleep bouts was unaffected (Fig. S1). Analysis of cumulative NREM time also implied some early NREM suppression immediately post-dosing (Fig. 1H). CBN but not zolpidem increased REM sleep onset latency (Fig. 1K, N). CBN had biphasic effects on % REM sleep; it initially suppressed % REM, before it increased % REM at ZT17-18 (Fig. 1L). Zolpidem, however, did not affect % REM sleep (Fig. 1O). CBN initially decreased REM sleep bout duration and REM sleep bout number (Fig. S1). However, CBN 10 mg/kg then increased REM sleep bout duration and REM sleep bout number at ZT18. Both CBN and zolpidem decreased total wake by reducing % active but not % quiet wake at time points corresponding with increased sleep (Fig. 1P–T, Fig. S1).

EEG power spectra were examined in response to CBN and zolpidem administration (Fig. S2A–D). Neither CBN nor zolpidem had robust effects on NREM delta power, although there was an increase at the 30 mg/kg and 100 mg/kg CBN doses at ZT17 and 18, and for 10 mg/kg zolpidem at ZT13 and 14 (Fig. S2A, B). Both CBN (all doses) and zolpidem increased REM theta power, with an immediate onset of action for zolpidem but a delayed onset for CBN (Fig. S2C, D). CBN and zolpidem both reduced locomotor activity coinciding with the pro-sleep effects of the compounds, although both compounds had little effect on body temperature (Fig. S2C, D). Indeed, the rats did not display any overt behavioral effects like the locomotor suppression and profound hypothermia that has been observed following administration of equivalent doses of Δ^9^-THC.

### The effects of repeated CBN exposure on sleep were subject to a degree of tolerance

**Fig. 2:**
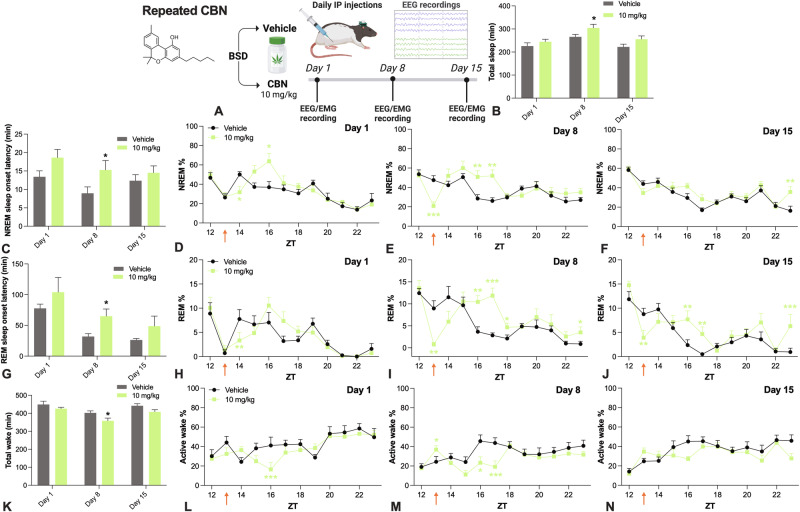
Effects of repeated CBN exposure on sleep and wake in rats. **A** Study design. CBN increased **B** total sleep time over days (CBN main effect, χ^2^_1_ = 5.291, *P* = 0.021; there was no CBN treatment by day interaction, inconsistent with tolerance). CBN increased **C** NREM sleep onset latency (CBN main effect, χ^2^_1_ = 5.632, *P* = 0.018). The effects of repeated CBN on % NREM on days **D** 1, **E** 8, and **F** 15 were subject to a degree of tolerance (CBN main effect, χ^2^_1_ = 7.946, *P* = 0.005, CBN and day interaction, χ^2^_2_ = 8.48, *P* = 0.014). CBN increased **G** REM sleep onset latency (CBN main effect, χ^2^_1_ = 4.073, *P* = 0.044). CBN affected % REM over days **H** 1, **I** 8, and **J** 15 (CBN main effect, χ^2^_1_ = 14.822, *P* = 0.0001; CBN and time interaction, χ^2^_10_ = 100.778, *P* < 0.0001). The effects of repeated CBN on % REM sleep were different over days (CBN, time and day interaction, χ^2^_20_ = 47.475, *P* = 0.001). Repeated CBN decreased **K** total wake time (CBN main effect, χ^2^_1_ = 5.467, *P* = 0.019, but no CBN and day interaction). CBN decreased % active wake over days **L** 1, **M** 8, and **N** 15 (CBN and time interaction, χ^2^_10_ = 40.168, *P* < 0.0001), which was subject to tolerance (CBN, time and day interaction, χ^2^_20_ = 32.219, *P* = 0.041). Time is expressed relative to lights on (ZT). Dunn–Šidák corrected multiple comparisons test *P < 0.05, **P < 0.01, ***P < 0.001, ****P < 0.0001. Error bars display ± SEM, *n* = 8 per group. CBN cannabinol, BSD between-subjects design, EEG electroencephalography, EMG electromyography, IP intraperitoneal, NREM non-rapid eye movement sleep, REM rapid eye movement sleep, ZT zeitgeber time. Created with BioRender.com.

A repeated dosing study was conducted to determine whether tolerance developed to the sleep-altering effects of CBN. CBN 10 mg/kg was administered daily for 15 days and EEG/EMG recordings were made on days 1, 8, and 15 (Fig. 2A). We selected a 10 mg/kg CBN dose, as it was the lowest effective dose and had the most desirable sleep profile. Overall, CBN increased total sleep time over days, with the most pronounced effect being on day 8 of administration (Fig. 2B). Unlike our acute study, CBN failed to reach statistical significance on day 1 on this measure. This might be explained by the different designs used across studies; a within-subjects design was used in the acute CBN study, which has greater statistical power and equally distributes inter-individual variability across groups, whereas a between-subjects design was used here. CBN increased NREM and REM sleep onset latencies compared to vehicle on day 8, but not on days 1 or 15 (Fig. 2C, G). CBN increased % NREM initially on days 1 and 8, however this effect was diminished by day 15 of treatment (Fig. 2D–F). However, repeated CBN consistently increased NREM sleep bout duration and decreased NREM sleep bout number over days that were less subject to tolerance (there were no CBN, time, or day interactions) (Fig. S3). Like in the acute administration study, there was evidence for CBN having biphasic effects on NREM and REM sleep in the repeated dosing study, with initial decrements before increments 3–4 h post-dose (Fig. 2D–F, H–J). The effects of CBN on % REM sleep appeared to be maintained over the days of treatment, although there was a significant CBN, time, and day interaction, which may have reflected if anything, a diminished impact of CBN on % REM sleep on day 1 than on day 15. The effects of repeated CBN exposure on active wake were subject to tolerance with reduced effects by day 15 of exposure (Fig. 2L–N).

### Novel chemical syntheses of the major metabolites of CBN, 11-OH-CBN, and 11-COOH-CBN

**Fig. 3:**
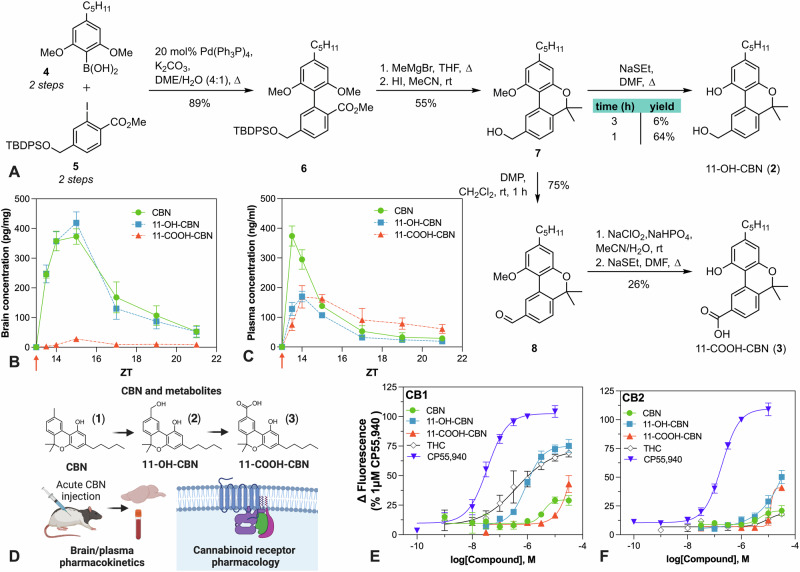
Total synthesis of CBN metabolites and their pharmacological characterization. **A** Synthetic route of primary metabolite 11-OH-CBN and terminal metabolite 11-COOH-CBN. **B** Brain and **C** plasma pharmacokinetic profile of CBN, 11-OH-CBN, and 11-COOH-CBN following administration of 10 mg/kg CBN IP to rats. 11-OH-CBN attained similar brain exposures to CBN. *n* = 4 per group. **D** Overview of pharmacological characterization of CBN and its major metabolites. Assessment of CBN and its metabolites at human cannabinoid **E** CB_1_ and **F** CB_2_ receptors expressed in AtT20 cells using membrane potential assay that reflects activation of G-protein-coupled inwardly rectifying potassium channels (GIRK). CBN showed low activity at CB_1_ receptors, whilst 11-OH-CBN behaved as a modestly potent partial agonist. Responses as depicted as a percentage of the response 1 µM CP 55,940, a potent, non-selective CB_1_/CB_2_ receptor agonist. *n* = 5–8 per group, performed in technical duplicate. CBN cannabinol, 11-OH-CBN 11-hydroxy-cannabinol; 11-COOH-CBN 11-carboxy-cannabinol, IP intraperitoneal. Created with BioRender.com.

The neuropharmacological effects of CBN (**1**) may derive, in part, from the in vivo formation of active metabolites. However, no prior study has evaluated the brain and plasma pharmacokinetics of the primary metabolite 11-hydroxy-CBN (11-OH-CBN, **2**) and the terminal metabolite 11-carboxy-CBN (11-COOH-CBN, **3**). Because no analytical standards for the metabolites were available at the time, (**2**) and (**3**) were synthesized *via* a new route taking inspiration from the total synthesis of related metabolite natural products Pulchrol and Pulchral [38] (Fig. 3A). The synthesis of 11-OH-CBN (**2**) commenced with Suzuki coupling of known boronic acid **4** [39] and aryl iodide **5** [40], each available in 2-steps, to give the corresponding methyl ester **6** in 87% yield. Next, the dual addition of methylmagnesium bromide to ester **6** followed by acid-mediated demethylation and cyclization proceeded smoothly, with concomitant silyl deprotection providing benzylic alcohol **7** in 55% yield. While demethylation of the second aryl methylether should be possible under these conditions, extended reaction times lead to decomposition, and therefore insufficient yields (<5%) of **2**. The final demethylation was achieved upon treatment with sodium ethanethiolate to furnish 11-OH-CBN (**2**), albeit, requiring careful control of the reaction time. Oxidation of 11-OH-CBN (**2**) to 11-COOH-CBN (**3**) was not feasible, either through direct means or *via* Pinnick oxidation of its corresponding aldehyde. However, its synthesis was achieved *via* oxidation of benzylic alcohol **7** to its corresponding aldehyde **8** with Dess-Martin periodinane (DMP), before Pinnick oxidation and finally demethylation.

### 11-OH-CBN attains high brain concentrations and is active at cannabinoid CB_1_ receptors

A pharmacokinetic study was then performed following the administration of CBN 10 mg/kg to rats and CBN and its metabolites 11-OH-CBN and 11-COOH-CBN were measured in brain and plasma (Fig. 3B, C, Table S1). Both CBN and the primary metabolite 11-OH-CBN had the same brain T_max_ (2 h), and the brain area-under-the-curve (AUC) and C_max_ of CBN and 11-OH-CBN were equivalent, suggesting the metabolite may contribute to the neuropharmacology of CBN. Both compounds were highly brain penetrant with brain-plasma ratios of 2.09 and 3.12 respectively. By contrast, the terminal metabolite 11-COOH-CBN was poorly brain penetrant (brain-plasma ratio = 0.12). The plasma T_max_ of CBN was 30 min, whereas the plasma T_max_ for 11-OH-CBN and 11-COOH-CBN was slightly delayed at 1 h post-dose. The half-life of CBN and 11-OH-CBN were similar in plasma and brain (approximately 2 h), whereas 11-COOH-CBN had a longer brain and plasma half-life (t_1/2_ plasma = 4.52 h, brain T_1/2_ = 3.48 h).

The effects of CBN and its metabolites were compared at human CB_1_ and CB_2_ receptors using the membrane potential assay (Fig. 3E, F). The responses were normalized to the potent non-selective CB_1_/CB_2_ agonist CP 55,940. We found that CBN induced a small response (maximal response (E_max_) = 29 ± 3%) with relatively low potency (pEC_50_ = 5.4 ± 0.1) (Fig. 3E) that was substantially less than Δ^9^-THC activity at CB_1_ (E_max_ = 72 ± 9%; pEC_50_ = 6.4 ± 0.3). CBN had negligible activity at CB_2_ (<20% at 10 µM). 11-OH-CBN also produced partial agonist activity at CB_1_ (E_max_ = 75 ± 3%) with a slightly lower potency than Δ^9^-THC (pEC_50_ = 6.0 ± 0.1) and some CB_2_ activity at higher concentrations (50 ± 6% at 30 µM), similar to previous reports using rat CB_1_ and human CB_2_ receptors [18]. 11-COOH-CBN activated both CB_1_ and CB_2_, but only at the highest concentration tested (30 µM; 43 ± 7% and 41 ± 2% at CB_1_ and CB_2_, respectively).

### Acute administration of 11-OH-CBN increased sleep in rats

**Fig. 4:**
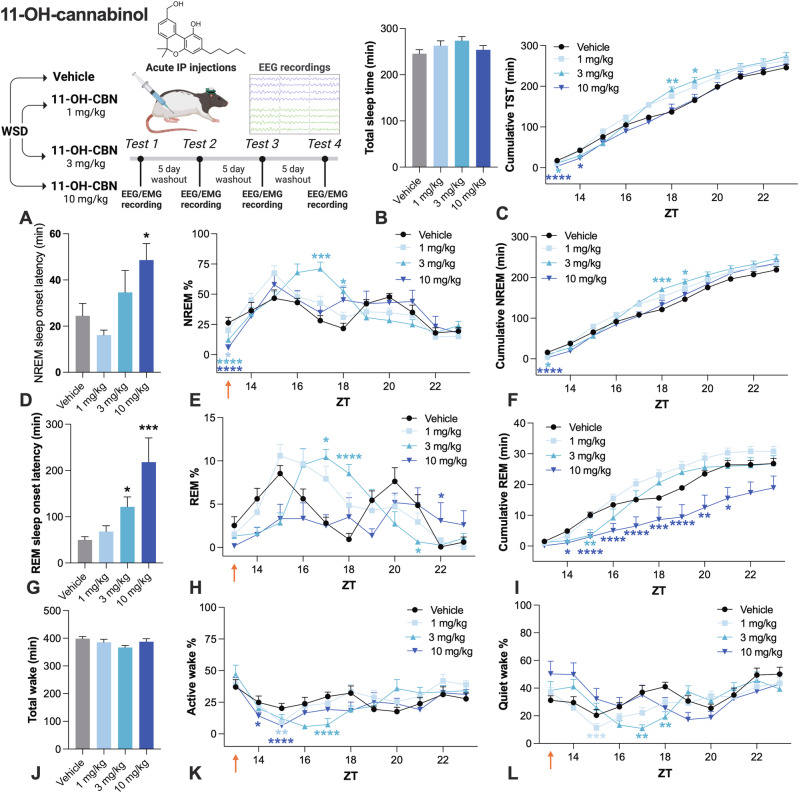
Effects of acute 11-OH-CBN on sleep and wake in rats. **A** Study design. 11-OH did not significantly increase **B** total sleep time, although it did increase **C** cumulative total sleep time, but in a biphasic manner with early-phase sleep suppression before subsequent enhancement (main effect 11-OH-CBN, χ^2^_3_ = 34.612, *P* < 0.0001, 11-OH-CBN and time interaction, χ^2^_30_ = 99.18, *P* < 0.0001). 11-OH-CBN significantly increased **D** NREM sleep onset latency (main effect 11-OH-CBN, χ^2^_3_ = 13.679, *P* = 0.003). **E** 11-OH-CBN increased % NREM (main effect 11-OH-CBN, χ^2^_3_ = 55.64, *P* < 0.0001, 11-OH-CBN and time interaction, χ^2^_30_ = 113.816, *P* < 0.0001). **F** CBN biphasically influenced cumulative total NREM sleep (main effect 11-OH-CBN, χ^2^_3_ = 36.884, *P* < 0.0001, 11-OH-CBN and time interaction, χ^2^_30_ = 107.499, *P* < 0.0001). 11-OH-CBN increased **G** REM sleep onset latency (main effect 11-OH-CBN, χ^2^_3_ = 26.885, *P* < 0.0001). 11-OH-CBN affected **H** % REM sleep (11-OH-CBN main effect, χ^2^_3_ = 9.676, *P* = 0.022; 11-OH CB and time interaction, χ^2^_30_ = 146.099, *P* < 0.0001). 11-OH-CBN suppressed **I** cumulative REM at the highest dose (11-OH-CBN and time interaction, χ^2^_30_ = 79.744, *P* < 0.0001). 11-OH-CBN did not affect **J** total wake, but decreased **K** % active wake (11-OH-CBN and time interaction, χ^2^_30_ = 94.753, *P* < 0.0001), and **L** % quiet wake (11-OH-CBN and time interaction χ^2^_30_ = 120.4587, *P* < 0.0001). Time is expressed relative to lights on (ZT). Dunn–Šidák corrected multiple comparisons test *P < 0.05, **P < 0.01, ***P < 0.001, ****P < 0.0001. Error bars display ± SEM, *n* = 8 per group. 11-OH-CBN 11-hydroxy-cannabinol, WSD within-subjects design (Latin square), EEG electroencephalography, EMG electromyography, IP intraperitoneal, NREM non-rapid eye movement sleep, REM rapid eye movement sleep, ZT zeitgeber time. Created with BioRender.

Given that 11-OH-CBN attained high brain concentrations and was pharmacologically active at cannabinoid CB_1_ receptors, we then examined whether it had pro-sleep effects using wireless polysomnography (Fig. 4A). While acute administration of 11-OH-CBN did not significantly increase total sleep time (Fig. 4B), it biphasically influenced cumulative total sleep time (Fig. 4C). Initially both the 3 and 10 mg/kg doses decreased total sleep time, but later the lower 1 and 3 mg/kg doses tended to increase cumulative total sleep time. 11-OH-CBN increased NREM and REM sleep onset latencies (Fig. 4A, D). 11-OH-CBN increased % NREM sleep, most robustly at 3 mg/kg with a delayed onset of action 3–4 h post-dose. Although 11-OH-CBN initially decreased % NREM compared to the vehicle at ZT13, it then increased % NREM at ZT17-18. This biphasic effect was reflected in cumulative NREM time (Fig. 4F). NREM sleep at ZT17-18 was characterized by an increase in the number of NREM sleep bouts and a decrease in the duration of NREM sleep bouts (Fig. S4). All doses increased NREM sleep bout number at ZT15. 11-OH-CBN biphasically affected % REM sleep, with initial suppression of % REM before a significant increase most pronounced at 3 mg/kg (Fig. H and I). The effects of 11-OH-CBN on % REM were driven by REM sleep bout number which followed a similar profile over time (Fig. S4). 11-OH-CBN did not significantly reduce total wake time (Fig. 4J). 11-OH-CBN decreased % active wake and % quiet wake (Fig. 4K, L). We also examined EEG power spectra in response to 11-OH-CBN administration (Fig. S5A, B). 11-OH-CBN increased NREM delta power, with robust effects observed at all doses at ZT14 (Fig. S5A). In addition, 11-OH-CBN increased REM theta power with effects across the recording window (Fig. S5B). 11-OH-CBN had a biphasic effect on locomotor activity, initially increasing activity at ZT13 but then decreasing activity ZT16-18 (Fig. S5C). 11-OH-CBN decreased body temperature with a delayed effect at ZT15-19 (Fig. S5D).

## Discussion

Products containing the minor plant cannabinoid CBN are being sold as sleep aids, but there is scant objective scientific evidence to support their use. Our results here justify further examination of CBN as a sleep therapeutic, by showing that CBN increases sleep based on objective measures determined by wireless polysomnography in rats. Acute administration of CBN to rats increased total sleep time by increasing NREM and REM sleep and decreasing wakefulness, although there was evidence of initial sleep suppression. CBN increased sleep stability as evidenced by longer duration NREM sleep bouts; this is significant as increased sleep stability has been associated with improved subjective sleep quality [41]. CBN’s effects on sleep were initially maintained following repeated, daily dosing but were subject to a degree of tolerance. CBN and its major primary metabolite, 11-OH-CBN, attained equivalently high brain concentrations, opening the possibility that 11-OH-CBN might contribute to the soporific effects of CBN. Examination of CBN and its metabolites on cannabinoid CB_1_ receptors, which are known to influence sleep, revealed CBN and the terminal metabolite 11-COOH-CBN had minimal activity, but the primary metabolite 11-OH-CBN behaved as a partial agonist, eliciting similar potency and efficacy to Δ^9^-THC in the membrane potential assay. We then explored the effects of 11-OH-CBN on sleep and found that it was active and influenced sleep architecture.

The overall results justify further investigation of the effect of CBN on sleep in preclinical and clinical studies. Recent classifications of sleep disorders include the common phenotypes of sleep onset insomnia, sleep maintenance insomnia, and early morning awakening insomnia. Our results here suggest that CBN might be best targeted to patients with sleep maintenance insomnia or early morning awakening insomnia, as CBN had a delayed onset of pro-sleep action that had a longer duration of action than zolpidem. Our study is unique because we provide data on the effects of CBN as a single molecule. Most human studies that have assessed CBN’s effects on sleep combined CBN with other phytocannabinoids, and have failed to assess whether CBN has hypnotic effects alone [26, 42]. The best human study to date assessed the effects of 20 mg CBN alone on sleep using self-reported measures in a non-clinical sample of poor sleepers [26]. Akin to the present results, CBN did not influence sleep onset latency but significantly reduced the number of awakenings and overall sleep disturbance compared to placebo [26]. However, this study did not assess multiple doses of CBN or its effects on objective measures such as polysomnography. Notably, our research group is conducting such a trial in primary insomnia patients [43]. In this study a 30 mg and 300 mg dose of purified CBN will be administered; the lower dose reflects the typical dose that is used in the community in CBN “isolate” products [43]. The higher dose of 300 mg was selected based on interspecies conversion from our 10 mg/kg CBN dose to a human equivalent dose (∼100 mg in a 60 kg human) [44], then increased by a factor of 3 to account for the low oral bioavailability of CBN. Indeed, CBN administered orally in rats yielded ∼5-fold lower plasma exposures (AUC) than that observed in the current study where CBN was administered intraperitoneally at the same dose [45]. The doses used in our study extrapolate to being much higher than that used in typical CBN isolate products available on the market. Moreover, the doses tested here are even less relevant to CBN doses administered via the smoking of Δ^9^-THC-dominant cannabis [46–48]. The highest plasma CBN concentration following cannabis smoking we found in the literature was a C_max_ of 11.6 ng/ml [47], which is dwarfed by the C_max_ of 374 ng/ml observed in our study following a 10 mg/kg dose in rats.

It is interesting to contrast our findings here with pharmacological studies that have addressed the role of central cannabinoid CB_1_ receptors in sleep. Our results agree with research showing that Δ^9^-THC, a partial agonist of the CB_1_ receptor, suppressed REM in rats [49, 50]. This has been a consistent finding across species, including humans [51–53]. Similar to our findings, rabbits administered Δ^9^-THC showed early-phase NREM and REM suppression, followed by a later phase increase in NREM sleep [51]. In an early human study, Δ^9^-THC tended to increase NREM but suppress REM sleep [53]. The effects of CBN and 11-OH-CBN reported here also broadly accord with sleep studies of cannabinoid tool molecules. The selective, full agonist of the CB_1_ receptor, CP 47,497, increased NREM in mice [21]. Further, AM3506 and JZL184, which increase brain levels of the endocannabinoids anandamide and 2-AG respectively via inhibition of degradative enzymes, both increased NREM and suppressed REM [21].

The present results provide the first evidence that the hypnotic effects of CBN may involve neuropharmacological actions of its primary metabolite 11-OH-CBN. Studies using hepatic microsomes reported that CBN forms various monohydroxy metabolites, the most prominent being 11-OH-CBN [54, 55]. Our results provide unprecedented evidence that following systemic administration of CBN, 11-OH-CBN attains similar micromolar concentrations to CBN in the brain (C_max_ = 1.3 µM and 1.2 µM respectively) and has a slightly higher brain/plasma ratio than CBN (B/P = ∼3 versus 2) (see Table S1 for full report on pharmacokinetic parameters). Moreover, whilst CBN had low activity at human CB_1_ receptors, 11-OH-CBN was active with notable partial agonist activity. This novel finding highlights that 11-OH-CBN has potency and efficacy similar to Δ^9^-THC at central CB_1_ receptors but is less active and efficacious than the synthetic cannabinoid receptor agonist CP 55,940. An earlier study using an adenylyl cyclase inhibition assay showed that 11-OH-CBN is active at rat CB_1_ receptors, reporting a higher potency than CBN (low nM range), but its efficacy was not reported [18]. Overall, the present data highlight that the 11-OH-CBN metabolite is active and may play an important role in the neuropharmacological effects of CBN in vivo. 11-OH-CBN was also active, albeit with subtle differences in its effects on sleep architecture to CBN. When considering cumulative total sleep time, there was a tendency for 11-OH-CBN to suppress sleep initially, before increasing the accumulation of total sleep time, which was similar to the profile observed with CBN. Both compounds increased % NREM, but one notable difference was that CBN increased the duration of NREM bouts and decreased the number of NREM bouts, whereas the opposite profile was observed for 11-OH-CBN on these parameters.

This is the first study to show that CBN and its active metabolite 11-OH-CBN influence sleep architecture, suggesting that these compounds or their derivatives could be further advanced in hypnotic drug discovery and development programs. Further examination in preclinical models of disordered sleep is necessary to help clarify which populations could be best targeted by the cannabinols. Given the biphasic effects observed, it would be interesting to explore whether the cannabinols shift circadian rhythm, as has been shown for other cannabinoids [56]. Whilst there is some overlap between the kinetics of the elevated brain concentrations of CBN and 11-OH-CBN and the increased NREM and REM sleep observed, we cannot rule out that these latter phase effects are simply explained by an adaptive rebound to NREM and REM suppression, as opposed to a direct pharmacological action of the compounds 3–4 h post-drug administration. The early-phase NREM and REM sleep-suppressing effects of CBN and 11-OH-CBN on REM sleep might be problematic for implementing CBN as a therapeutic, particularly as REM sleep suppression impairs memory consolidation, and thus the cannabinols might have deleterious effects on cognitive function [57]. Although CBN and 11-OH-CBN both increased REM theta power which might improve memory consolidation [58]. Future studies are needed to observe whether the effects of the cannabinols on sleep translate into any effects on cognitive function.

There are several limitations to the current study that warrant discussion. First, we have not unequivocally proven that CB_1_ receptors mediate the pro-sleep effects of the cannabinols. This is because studies using selective receptor antagonists or gene knockout would be very challenging, given that both these manipulations are not silent and affect sleep themselves [20, 21]. It is also possible the effects are explained by other molecular targets of CBN such as transient receptor potential (TRP) channels like TRPA1 and TRPV2, which may influence circadian rhythms and sleep-wake cycles [14, 59–62]. Additional limitations are that the study only examined male rats and that the cannabinols were only administered in the dark phase. Thus, future studies are needed to confirm activity in females and in the light phase. Whilst the study addressed the impact of repeated CBN exposure on sleep, it did not examine whether withdrawal of CBN had any detrimental effects on sleep. Cannabis withdrawal is associated with insomnia and sleep disruption following the withdrawal of cannabinoid receptor agonists [63, 64]. A future study could examine whether CBN withdrawal adversely impacts sleep.

In conclusion, for close to 50 years there has been the suggestion that the minor plant cannabinoid CBN increases sleep without any robust scientific evidence based on objective sleep measures. This study provides the first objective evidence that CBN influences sleep architecture and that its hypnotic effects may involve an active metabolite. Whilst the study encourages further development of the cannabinols for sleep, it also underscores the need for a nuanced approach that takes heed of the narrow dosing window for pro-sleep effects and the complex, biphasic activity the cannabinols have on sleep.

## Supplementary information

Supplementary material

## Data Availability

All data are available via the Dryad Digital Repository.
