# Supplementary material for: A sleepy cannabis constituent: cannabinol and its active metabolite influence sleep architecture in rats
Source: Neuropsychopharmacology. 2024 Nov 12;50(3):586–95. doi: 10.1038/s41386-024-02018-7 (PMC11736144; doi:10.1038/s41386-024-02018-7)
Supplement: Supplementary file 1 — Supplementary material [file 41386_2024_2018_MOESM1_ESM.docx]

**Supplemental Material.**

**Materials and Methods.**

**Drugs.** The CBN doses tested encompass those that have been shown to promote cannabimimetic effects such as reduced locomotor activity, hypothermia, as well as doses known to potentiate pentobarbital-induced sleep time [1,2]. Although, it should be noted that there was conflicting data reported in these studies. Järbe et al (1987) reported that 10 and 30 mg/kg CBN IP reduced body temperature and suppressed locomotor activity in rats. While the CBN used was 99.2% pure, there was an impurity of 0.8% Δ^8^-THC, which might have contributed to the effects observed. Inconsistent with this Fernandes et al (1974) reported that CBN up to 80 mg/kg IP did not influence body temperature or locomotor activity; the CBN product was 98% pure, with other minor cannabinoids being present but no report of the presence of Δ^8^-THC or Δ^9^-THC.

**Surgical implantation of biotelemetry probes.** Rats were administered 0.05 mg/kg buprenorphine subcutaneous (SC) and 0.5 mg/kg bupivacaine as preoperative anaesthesia prior to general induction with isoflurane for the duration of the surgery. After induction, animals were transferred to a stereotaxic frame where a small subcutaneous incision was made to the flank into which the probe was inserted. A cranial incision was then made allowing visualisation of lambda, bregma and trapezius neck muscles. Trepanations were made using a 1 mm stereotaxic drill at recording sites: 1.5 mm mediolateral and 2 mm anteroposterior to bregma and 1.5 mm mediolateral and 7 mm anteroposterior to bregma. Stainless-steel screws were inserted at the two sites around which the EEG leads were wound and securely insulated using Kwik-Sil ^TM^. The EMG leads were guided subcutaneously from the probe and wires were threaded through the trapezius neck muscle. Incisions were then closed with non-absorbable sutures.

Immediately post-surgery the rats were administered the anti-inflammatory and analgesic agent meloxicam at 2 mg/kg and then every 24 h for two days. Following recovery, rats were returned to group housing where they remained for 2 weeks. Rats were monitored daily and were healthy before the commencement of baseline recordings. At the completion of all treatments, rats were euthanised by transcardial perfusion. Lethabarb was administered prior to perfusion with phosphate-buffered saline before 4% paraformaldehyde fixative. Brains were collected for verification of EEG electrode placement.

**Analysis of sleep recordings.** Filters were applied to polysomnographic data. A band pass filter (0.1-80 Hz) was applied to EEG data to limit signal to relevant bandwidths and a high pass filter (> 0.1 Hz) was applied to EMG data to exclude irrelevant low frequency activity and reduce random noise. EEG was passed through Fast Fourier transformation allowing visualisation of temporal changes in spectral patterns of component frequencies. Distinct vigilance states were quantified based on power band density, frequency and amplitude of EEG, EMG and activity counts.

Recordings were scored as 10 s epochs and all measures were averaged into 1 h time bins for analysis. If no signal was present for > 50% of the epoch it was assigned as an artifact. A vigilance state bout was assigned to an epoch if it contained characteristics of that stage for > 50% of the 10 s period. An epoch was scored as active wake if locomotor activity was present in the absence of EEG data, and if it was consistent with the adjacent epochs.

The scoring protocol was adapted from Neckelmann and Ursin (1993) and vigilance states were labelled as bouts and their duration measured manually as follows [3]:

1. *Active wake* was defined as a period of physical arousal featuring irregular, high-amplitude EMG tone often corresponding with locomotor activity. EEG signal was low amplitude (< 80 μV), highly asynchronous and periodically marked by increased theta activity (4-8 Hz).
2. *Quiet wake* was often characterized by a significant reduction in physical arousal compared to active wake, noted by reduced EMG voltage and regular profile which corresponded with an absence of activity counts.
3. *NREM sleep* was marked by increased amplitude and reduced EEG frequency (approximately 250-500 μV) corresponding with a notable rise in delta activity (0.5-4 Hz) relative to quiet wake. EMG amplitude was further reduced from quiet wake.
4. *REM sleep* was characterized by low amplitude (< 80 μV), high frequency, EEG
   activity. The EEG of REM sleep resembles wake but can also be recognized by the increased prominence of lower frequency waveforms such as theta and sawtooth waves giving the EEG a more jagged form. EMG tone was virtually absent, allowing for a further distinction from wake.

From this scoring the following sleep measures were extracted: NREM or REM sleep onset latency (the number of seconds to reach the first NREM or REM sleep epoch following the return of the rat to its cage after injection, min); % NREM or REM sleep (the proportion of time spent in each sleep state); NREM and REM bouts (frequency of scored sleep events); NREM or REM bout duration (the mean duration of each bout, min); and total sleep time (TST) (combined NREM and REM bout duration, min).

*EEG power spectra* data were processed using Fast Fourier transformation in 10 s epochs with Neuroscore software. Delta (1-4 Hz) and theta (4-8 Hz) power densities were calculated for each epoch. The data were filtered according to vigilance state, and power densities for delta and theta were analyzed for only epochs classified as NREM or REM sleep, respectively. Data were averaged into one hour bins resulting in missing values for animals that did not experience NREM or REM sleep within a given hour.

**LC-MS/MS.** Plasma samples (50 µL) were spiked with internal standard (12.5 µg/mL d_3_-THC, 5 µL) and protein precipitation was achieved by vortex-mixing with a 3x volume of ice-cold acetonitrile. The organic layer was isolated by centrifugation (6000 g for 10 min, 4 °C) and evaporated to dryness using N_2_ gas. Samples were reconstituted in acetonitrile (90 µL) and 0.1% ammonium formate in water (300 µL) for supported-liquid extraction (SLE) using dichloromethane and methyl *tert*-butyl ether Biotage Isolute SLE+ columns (Uppsala, Sweden) as previously described [4]. Brain samples were prepared as described for plasma samples with minor modifications. Briefly, each half brain was homogenised in methanol: water (2:1, 10x, w/v) and spiked with d_3_-THC as an internal standard. Homogenates were centrifuged (20,000 g for 30 min, 4 °C). Supernatants were then extracted and prepared as described above.

Quantification of CBN, 11-OH-CBN and 11-COOH-CBN in plasma and brain samples was performed by comparing experimental samples to eight-point standard curves prepared with known amounts of cannabinoid. Plasma and brain concentrations at each time point were averaged and pharmacokinetic parameters were calculated by noncompartmental analysis. Elimination rate constants were determined by linear regression of the terminal component of the concentration-time curve using GraphPad Prism 8.2 (La Jolla, USA). The log-linear trapezoidal method was used to calculate the area under concentration-time curve using equations described previously [5].

***In vitro* functional characterization at human CB_1_ and CB_2_ receptors.** Briefly, mouse AtT20 FlpIn adenocarcinoma cells stably transfected with human CB_1_ or CB_2_ receptors were cultured in DMEM containing 10% FBS, 100 U penicillin/streptomycin, and 80 μg/mL of hygromycin. Cells were passaged at 80% confluency in 75 cm^2^ flasks, and used at 90% confluence. A day before the assay, cells were detached from the flask with trypsin/EDTA (Sigma Aldrich) and resuspended in 10 mL of Leibovitz's L-15 media supplemented with 1% FBS, 100 U penicillin/streptomycin, and 15 mM glucose ("L-15"). The cells were plated in black-walled, clear-bottomed 384-well (22.5 μL) microplates (Corning, Corning, NY, USA) and incubated overnight at 37°C with ambient CO_2_. A Membrane Potential Assay Kit (blue) from Molecular Devices (San Jose, CA, USA) was used to measure changes in membrane potential that reflects activation of G-protein-coupled inwardly rectifying potassium channels (GIRK). The dye was reconstituted (at 50% of manufacturer’s recommendation) with Hank’s Balanced Salt solution (HBSS) assay buffer [145 mM NaCl, 22 mM HEPES, 0.338 mM Na_2_HPO_4_, 4.17 mM NaHCO_3_, 0.441 mM KH_2_PO_4_, 0.407 mM MgSO_4_, 0.493 mM MgCl_2_, 1.26 mM CaCl_2_, 5.56 mM glucose (pH 7.4)], loaded 1:1 with L-15 in each well, 1 hour prior to assay initiation. Fluorescence was measured using a FlexStation 3 (Molecular Devices) microplate reader (λ_ex_ = 530 nm and λ_em_ = 565 nm). Baseline readings were taken every 2 s for 2 min, followed by compound addition (5 μL in 384-well plate) and read for 3 min. The background fluorescence of cells without dye or dye without cells was negligible. Changes in fluorescence from baseline were normalized to a positive control (1 μM CP 55,940) after subtraction of fluorescence changes produced by the vehicle alone (0.1% DMSO with 0.01% BSA in assay buffer). Concentration-response data were analyzed using GraphPad Prism 9 (GraphPad Software, San Diego, CA, USA), using a four-parameter non-linear regression on data from 5 to 8 independent experiments performed in technical duplicate.

**Medicinal chemistry.** All reactions were performed under an atmosphere of nitrogen unless otherwise specified. Deuterated solvents (CDCl_3_, and DMSO-*d_6_*) were purchased from Cambridge Isotope Laboratories (Tewksbury, MA, USA). Unless otherwise stated, all other reagents and solvents used were obtained from Sigma-Aldrich/Merck (Castle Hill, NSW, Australia) and used as purchased. Analytical thin-layer chromatography was performed using Merck aluminium-backed silica gel 60 F254 (0.2 mm) plates (Merck, Darmstadt, Germany), which were visualized using shortwave (254 nm) UV fluorescence. Flash chromatography was performed using a Biotage Isolera Spektra One and Biotage SNAP KP-Sil silica cartridges (Uppsala, Sweden). Nuclear magnetic resonance (NMR) spectra were recorded at 298 K using an Agilent 400-MHz spectrometer (Santa Clara, CA, USA), or a Bruker NEO 300 MHz NMR spectrometer (Billerica, MA, USA). The data are reported as chemical shift (*δ* ppm) relative to the residual protonated solvent resonance, relative integral, multiplicity (s = singlet, brs = broad singlet, d = doublet, t = triplet, q = quartet, quin. = quintet, m = multiplet), coupling constants (*J* Hz), and assignment. High-resolution mass spectrometry (HRMS) data were collected using an Agilent LC 1260-QTOF/MS 6550 (Santa Clara, CA, US) or a Bruker Solarix 2xR 7T Fourier Transform Ion Cyclotron Resonance Mass Spectrometer. A methanolic extract of each pure standard was run using an electrospray ionization source in automated MS/MS (information dependent acquisition) mode. Accurate mass for the parent ion and its corresponding mass error expressed in parts per million (ppm) are reported. LC-MS/UV data was acquired using a Shimadzu (Kyoto, Japan) system described above. Compounds were dissolved in a 90:10 mixture of 0.1% formic acid in water and methanol, and placed in the UHPLC autosampler that was maintained at 8 °C. Elution was performed in gradient mode with an Agilent (CA, USA) Zorbax XDB-C18 column (2.1 x 50 mm, 3.5 µm particle size), held at 50 °C, with a 10 µL injection volume. The mobile phases were 0.1% formic acid in water (A) and methanol (B). Mobile phase composition was held at 10% B until 0.5 min, then steadily increasing to 100% B at 2.5 min, holding until 3 min, and then re-equilibrating at 10% B for a total run time of 4 min. UV absorbance was measured from 190 to 800 nm. UV data from blank injections were subtracted to account for mobile phase absorbance and background noise. Single stage mass spectra (MS1) were collected in positive ESI mode using a single quadrupole (Q3) scanning from *m/z* 100 – 600. All data was processed using Shimadzu LabSolutions (v 5.89) software. Fourier-transform infrared spectroscopy (FTIR) spectra were collected using a Bruker Alpha II ATR FTIR spectrophotometer. Synthetic procedures and characterization data (^1^H and ^13^C NMR, R*_f_*, FTIR, HRMS, and LCMS) can be found in the supporting information.

**(2,6-dimethoxy-4-pentylphenyl)boronic acid (4).** The title compound was prepared according to the procedure reported by Yamada [6] using 1,3-dimethoxy-5-pentylbenzene (9.60 g, 46.1 mmol) and was isolated as a white crystalline solid (10.0 g, 86% yield). ^1^H NMR (400 MHz, DMSO-*d*6) δ 7.71 (s, 2H), 6.39 (s, 2H), 3.68 (s, 6H), 2.55 – 2.51 (m, 2H), 1.61 – 1.52 (m, 2H), 1.39 – 1.22 (m, 4H), 0.87 (t, *J* = 6.8 Hz, 3H). Analytical data was consistent were that reported previously [7]

**methyl 4-(((tert-butyldiphenylsilyl)oxy)methyl)-2-iodobenzoate (5).** The title compound was prepared according to the procedure reported by Sterner [8], and was isolated as a white amorphous solid (82% yield). 1 H NMR (400 MHz, chloroform-d 7.95 (s, 1H),7.80 (d, J = 8.0 Hz, 1H), 7.72 -7.62 (m, 7H), 4.73 (s, 2H), 3.93 (s, 3H), 1.11 (s, 9H). Analytical data were consistent with those reported previously [8].

**methyl 5-(((tert-butyldiphenylsilyl)oxy)methyl)-2',6'-dimethoxy-4'-pentyl-[1,1'-biphenyl]-2-carboxylate (6).** An RBF was charged with ethyl methyl 4-(((tert-butyldiphenylsilyl)oxy)methyl)-2-iodobenzoate (**5**) (900 mg, 1.7 mmol, 1 equiv.), (2,6-dimethoxy-4-pentylphenyl)boronic acid (**4**) (453 mg, 1.8 mmol, 1.06 equiv.), potassium carbonate (938 mg, 6.8 mmol, 4 equiv.), Pd(PPh_3_)_4_ (392 mg, 0.34 mmol, 0.2 equiv.) dimethoxyethane (31 mL) and water (6 mL). The mixture was immediately degassed *via* 3 x freeze/pump/thaw cycles before being heated to 100 °C for 16 hours. After this time, the solution was cooled to room temperature, filtered through a pad of celite, and the solvent removed under reduced pressure. The resulting crude oil was purified by flash chromatography using a Biotage Isolera One (1-20% EtOAc/hexane) to give the title compound (905 mg, 87% yield) as yellow oil. R*_f_* = 0.41 (10% EtOAc/hexanes); ^1^H NMR (400 MHz, CDCl_3_) δ 7.91 (d, *J* = 8.0 Hz, 1H), 7.75 – 7.66 (m, 4H), 7.48 – 7.32 (m, 8H), 6.46 (s, 2H), 4.82 (s, 3H), 3.69 (s, 5H), 3.64 (s, 2H), 2.71 – 2.59 (m, 2H), 1.74 – 1.65 (m, 2H), 1.42-1.34 (m, 4H), 1.10 (s, 9H), 0.93 (t, *J* = 7.1 Hz 3H); ^13^C NMR (101 MHz, CDCl_3_) δ 168.4, 156.9, 144.3, 144.1, 135.7, 135.1, 133.5, 130.5, 130.3, 129.8(4), 129.8, 129.8, 127.9, 124.3, 116.5, 104.4, 65.3, 55.9, 51.6, 36.9, 31.8, 31.2, 26.9, 22.7, 19.5, 14.2; HRMS (ESI, +ive) C_38_H_46_O_5_Si theoretical mass 610.3115, experimental exact mass 610.3117 (mass error 1.51 ppm).

**(1-methoxy-6,6-dimethyl-3-pentyl-6H-benzo[c]chromen-9-yl)methanol (7).** To a cooled (0 °C) solution of methyl 5-(((tert-butyldiphenylsilyl)oxy)methyl)-2',6'-dimethoxy-4'-pentyl-[1,1'-biphenyl]-2-carboxylate (**6**) (570 mg, 0.93 mmol, 1 equiv.) in THF (20 mL) was added MeMgI (1.24 mL, 3 M in Et_2_O, 3.73 mmol, 4 equiv.) dropwise. The mixture was heated to reflux and stirred for 16 hours before being quenched with NH_4_Cl (20 mL of saturated aqueous solution). The mixture was extracted with EtOAc (3 x 20mL), and the combined organics washed brine (2 x 20 mL) before being dried (Na_2_SO4), filtered, and concentrated under reduced pressure. The crude yellow residue was dissolved in acetonitrile (15 mL) and HI (0.30 mL, 55 wt% in H_2_O) added. The resulting solution was stirred for 3 hours before being quenched by the addition of sodium thiosulfate (20 mL of a saturated aqueous solution). The aqueous layer was extracted with EtOAc (3 x 30 mL) and the organics dried (Na_2_SO4), before being filtered, and concentrated under reduced pressure. The crude material was purified *via* flash column chromatography using Biotage Isolera One (5-40%) to afford the title compound (176 mg, 55% yield) as a yellow oil. R*_f_* = 0.22 (20% EtOAc/hexanes); ^1^H NMR (400 MHz, CDCl_3_) δ 8.40 (d, *J* = 1.7 Hz, 1H), 7.28 – 7.21 (m, 2H 6.46 (dd, *J* = 15.4, 1.6 Hz, 2H), 4.71 (s, 2H), 3.94 (s, 3H), 2.60 – 2.51 (m, 2H), 1.66 – 1.61 (m, 1H), 1.60 (s, 6H), 1.37 – 1.30 (m, 4H), 0.93 – 0.84 (m, 3H); ^13^C NMR (101 MHz, CDCl_3_) δ 157.6, 154.5, 144.9, 139.8, 139.2, 128.3, 125.7, 125.5, 122.9, 111.0, 109.7, 105.2, 77.3, 65.9, 55.7, 36.3, 31.7, 30.8, 27.1, 22.7, 14.2; HRMS (ESI, +ive) C_22_H_28_O_3_ theoretical mass 340.2038, experimental exact mass 340.2037 (mass error 1.01 ppm).

**9-(hydroxymethyl)-6,6-dimethyl-3-pentyl-6H-benzo[c]chromen-1-ol (11-OH-CBN, 2).** To a cooled (0 °C) suspension of sodium hydride (125 mg, 60% dispersion in mineral oil, 3.14 mmol, 10 equiv) in DMF (3 mL) was added ethanethiol (227 uL, 3.14 mmol, 10 equiv.) dropwise. The mixture was stirred for 30 minutes before being added *via* syringe to a solution of (1-methoxy-6,6-dimethyl-3-pentyl-6H-benzo[c]chromen-9-yl)methanol (**7**) (107 mg, 0.314 mmol, 1 equiv.) in DMF (2 mL). The resulting solution was heated to 140 °C and stirred for 1.5 hours before being cooled to room temperature and quenched by the addition of NaHCO_3_ (10 mL of a saturated aqueous solution). The aqueous layer was extracted with EtOAc (3 x 15 mL) and the organics washed with water (3 x 15 mL) and brine (15 mL). The combined organics were dried (Na_2_SO4), filtered, concentrated under reduced pressure and the crude residue purified *via* flash column chromatography using Biotage Isolera One (5-40% EtOAC/hexanes) to afford a yellow solid. The solid was taken up in a minimum amount of ethyl acetate and dichloromethane was added until white needles had formed. The solids were filtered and washed with cold dichloromethane to give the title compound (66 mg, 64% yield) as fine white needles. ^1^H NMR (400 MHz, CDCl_3_) δ 9.91 (brs, 1H, ArOH), 8.42 (d, *J* = 1.7 Hz, 1H), 7.24 (d, *J* = 7.9 Hz, 1H), 7.21 – 7.14 (m, 1H), 6.39 (d, *J* = 1.7 Hz, 1H), 6.24 (d, *J* = 1.7 Hz, 1H), 5.14 (t, *J* = 5.1 Hz, 1H), 4.48 (d, *J* = 5.1 Hz, 2H), 2.43 (t, *J* = 7.6 Hz, 2H), 1.55 (p, *J* = 7.6 Hz, 2H), 1.50 (s, 6H), 1.37 – 1.21 (m, 4H), 0.87 (t, *J* = 6.9 Hz, 3H). Analytical data were consistent with those reported previously [9].

**1-methoxy-6,6-dimethyl-3-pentyl-6H-benzo[c]chromene-9-carbaldehyde (8).** To a solution of (1-methoxy-6,6-dimethyl-3-pentyl-6H-benzo[c]chromen-9-yl)methanol (**7**) (300 mg, 0.881 mmol, 1 equiv.) in CH_2_Cl_2_ (20 mL) at 0 °C was added Dess-Martin periodinane (486 mg, 1.15 mmol, 1.2 equiv.) in portions. The reaction was warmed to room temperature and stirred for 3 hours before NaHCO_3_ (20 mL of a saturated aqueous solution) was added. The aqueous was extracted with CH_2_Cl_2_(2 x 10 mL) and the combined organics were dried (Na_2_SO4), filtered, and concentrated under reduced pressure. The crude residue was purified *via* flash column chromatography using Biotage Isolera One (0-10% EtOAc/hexanes) to afford the title compound (228 mg, 75% yield) as a yellow oil. R*_f_* = 0.41 (10% EtOAc/hexanes); ^1^H NMR (400 MHz, CDCl_3_) δ 10.04 (s, 1H), 8.91 (d, *J* = 1.7 Hz, 1H), 7.76 (dd, *J* = 7.9, 1.7 Hz, 1H), 7.38 (d, *J* = 8.0 Hz, 1H), 6.49 (dd, *J* = 8.9, 1.5 Hz, 2H), 3.98 (s, 3H), 2.61 – 2.56 (m, 2H), 1.69 – 1.64 (m, 2H), 1.63 (s, 6H), 1.42 – 1.30 (m, 4H), 0.91 (t, *J* = 6.9 Hz, 3H); ^13^C NMR (101 MHz, CDCl_3_) δ 192.8, 157.7, 154.3, 145.8, 145.6, 135.6, 129.6, 129.2, 129.1, 127.5, 123.4, 111.0, 105.3, 55.8, 55.7, 36.4, 31.7, 30.8, 26.9, 22.7, 14.2 (one signal missing or overlapping);; HRMS (ESI, +ive) C_22_H_26_O_3_ theoretical mass 338.1882, experimental exact mass 338.1879 (mass error 1.42 ppm).

**1-hydroxy-6,6-dimethyl-3-pentyl-6H-benzo[c]chromene-9-carboxylic acid (11-COOH-CBN, 3).** To a solution of 1-methoxy-6,6-dimethyl-3-pentyl-6H-benzo[c]chromene-9-carbaldehyde (**8**) (230 mg, 0.679 mmol, 1 equiv.) and NaH_2_PO_4_ (123 mg, 1.36 mmol, 2 equiv.) in acetonitrile:water (5 mL, 3:2) was added dropwise NaClO_2_ (203 mg, 1.70 mmol, 2.5 equiv.) in water (0.5 mL). The solution was stirred for 16 at room temperature before HCl (5 mL of a 1 M aqueous solution) was added and the mixture extracted with EtOAC (3 x 20 mL). The combined organics were washed with water (2 x 20 mL) and brine (1 x 20 mL) before being dried (Na_2_SO_4_), filtered and concentrated under reduced pressure. The crude residue was subjected *via* flash column chromatography using Biotage Isolera One (0-10% MeOH/CH_2_Cl_2_) to afford impure intermediate

**1-methoxy-6,6-dimethyl-3-pentyl-6H-benzo[c]chromene-9-carboxylic acid** (**SI-1).** Made (115 mg) as a yellow oil which could not be purified further. ^1^H NMR (400 MHz, CDCl_3_) δ 9.16 (d, *J* = 1.7 Hz, 1H), 7.98 (dd, *J* = 8.1, 1.8 Hz, 1H), 7.33 (d, *J* = 8.1 Hz, 1H), 6.51 – 6.45 (m, 2H), 3.99 (s, 3H), 2.61 – 2.55  (m,  2H), 1.73 (p, *J* = 6.1 Hz, 2H), 1.63 (s, 6H), 1.44 – 1.34 (m, 4H), 0.95 (t, *J* = 7.5 Hz, 3H). The resulting yellow oil was dissolved in dry CH_2_Cl_2_ (3 mL) and cooled to 0 °C before boron tribromide (0.72 mL, 0.72 mmol, 2.2 equiv) was added dropwise. The solution was warmed to room temperature and stirred for 1 h before being quenched by the addition of NaHCO_3_ (5 mL of a saturated aqueous solution). The aqueous was extracted with CH_2_Cl_2_(3 x 10 mL) before the combined organics were dried (Na_2_SO4), filtered and concentrated under reduced pressure. The crude residue was purified *via* flash column chromatography using Biotage Isolera One (0-10% MeOH:CH_2_Cl_2_) to afford the title compound (31 mg, 26% yield) as clear colourless film. ^1^H NMR (400 MHz, CDCl_3_) δ 9.17 (d, *J* = 1.7 Hz, 1H), 7.98 (dd, *J* = 8.1, 1.8 Hz, 1H), 7.34 (d, *J* = 8.1 Hz, 1H), 6.45 (d, *J* = 1.5 Hz, 1H), 6.32 (d, *J* = 1.6 Hz, 1H), 2.58 – 2.45 (m, 2H), 1.64 (s, 6H), 1.63 – 1.56 (m, 2H), 1.37 – 1.29 (m, 4H), 0.89 (t, *J* = 6.9 Hz, 3H). Analytical data were consistent with those reported previously [9].

**^1^H and ^13^C NMR Spectra of new compounds (6, 7 and 8)**

| methyl 5-(((tert-butyldiphenylsilyl)oxy)methyl)-2',6'-dimethoxy-4'-pentyl-[1,1'-biphenyl]-2-carboxylate **(6)** | |
| --- | --- |
| 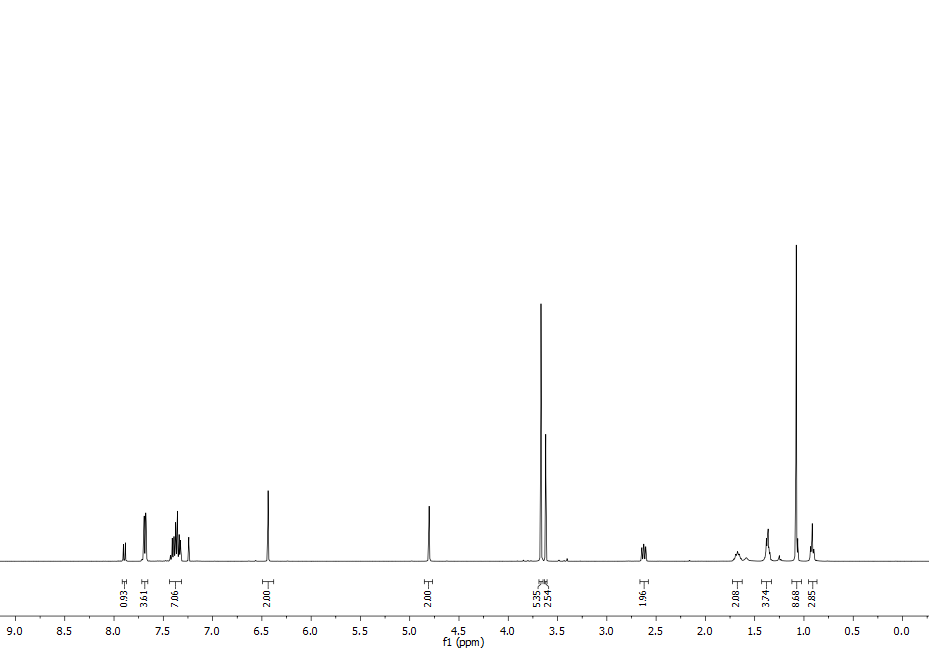 | 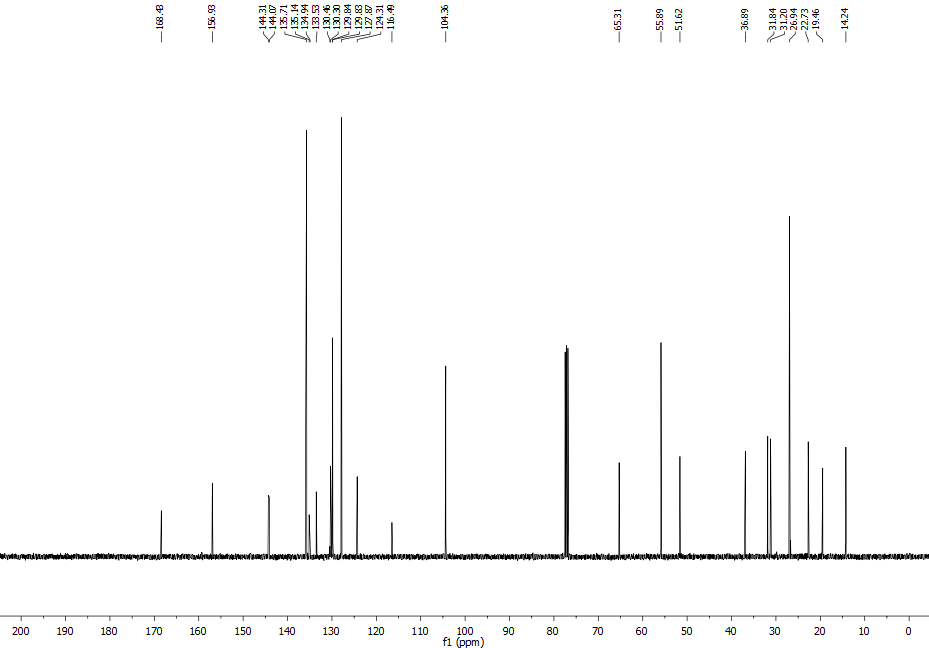 |

| (1-methoxy-6,6-dimethyl-3-pentyl-6H-benzo[c]chromen-9-yl)methanol **(7)** | |
| --- | --- |
| 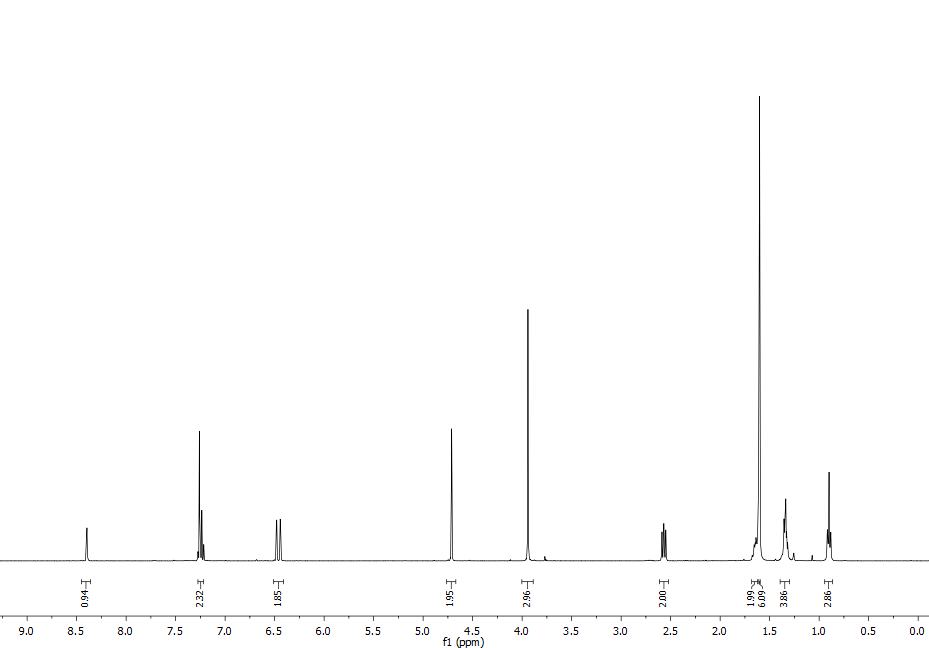 | 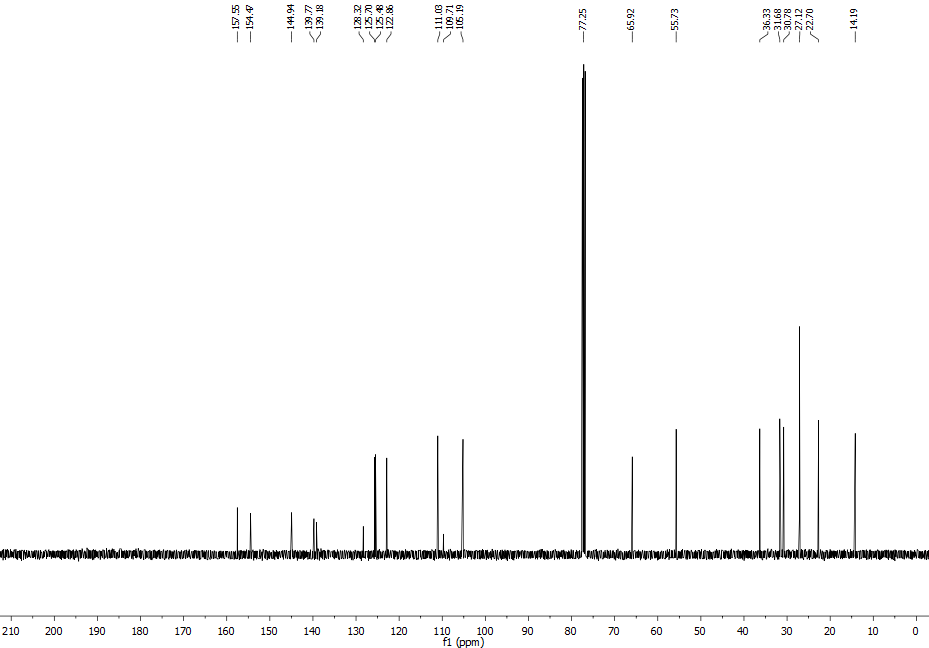 |

| 1-methoxy-6,6-dimethyl-3-pentyl-6H-benzo[c]chromene-9-carbaldehyde **(8)** | |
| --- | --- |
| 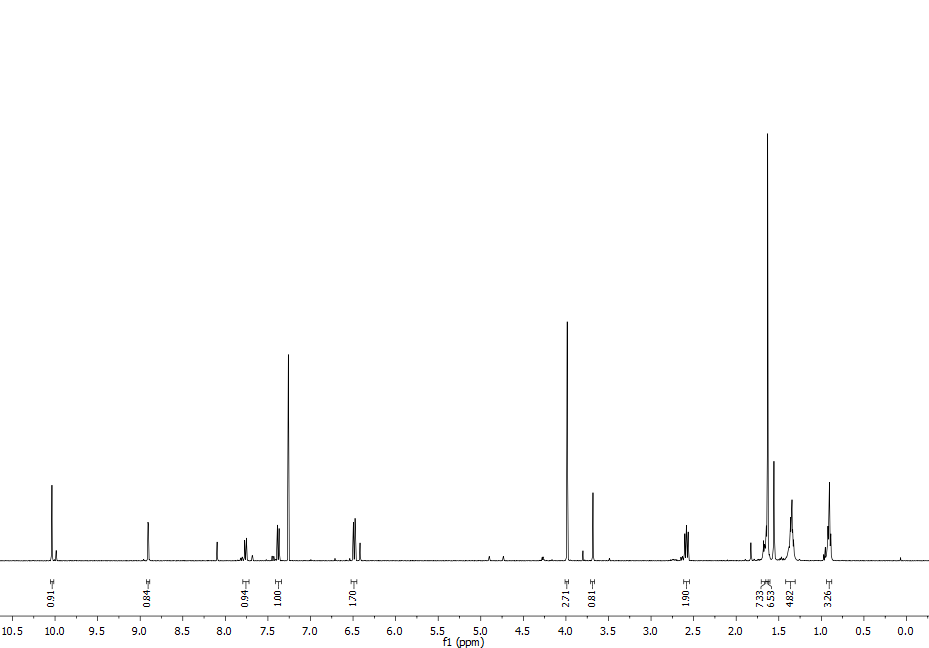 | 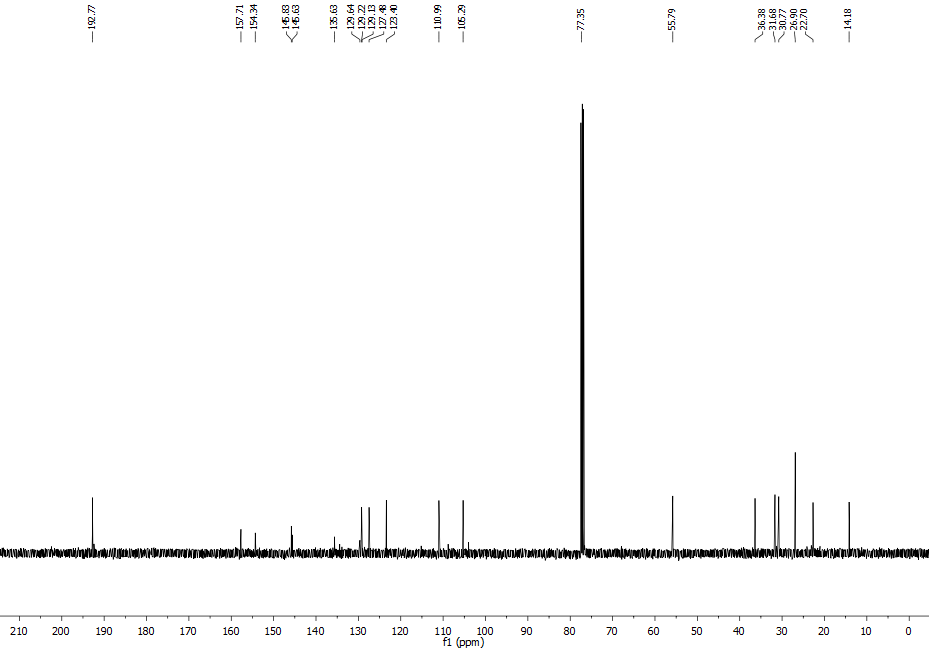 |

| 1-methoxy-6,6-dimethyl-3-pentyl-6H-benzo[c]chromene-9-carboxylic acid **(SI-1)** | |
| --- | --- |
| 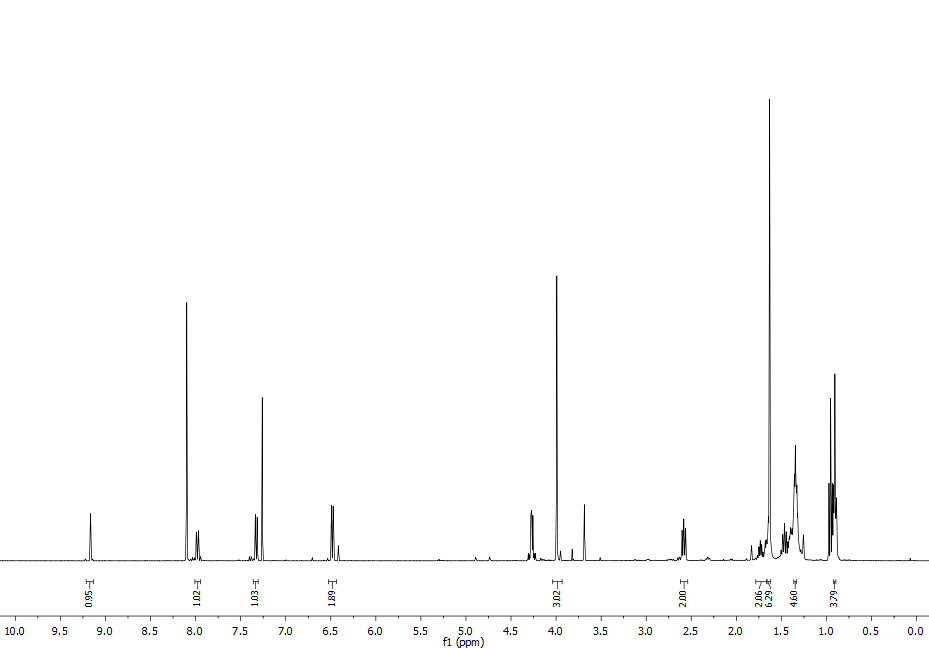 |  |

**Statistical analyses.** For both acute experiments, main effects of drug treatment, time or their interaction was assessed. For the repeated dosing experiment, main effects of treatment, time or day, and their double and triple interactions on measured variables was assessed. In all analyses, the identity of the individual was the only random factor to account for repeated measure effects. Model construction and fitting used the functions *lmer*, *glmer,* or *glmer.nb* from the package “lme4” version 1.1–30 [10] or the function *glmmTMB* from the package “glmmTMB” [11]. The data type (integers or continuous); residual plots; Shapiro–Wilk test of normality, using the function *shapiro.test* from the package “stats” [12]; Levene's test for homogeneity of variance using the function *leveneTest* from the package “car” [13]; and Pearson's dispersion test were used to identify the best distribution (i.e., gaussian, gamma, or negative binomial) and link (i.e., identity, inverse, log or square root) for each model [14].

**
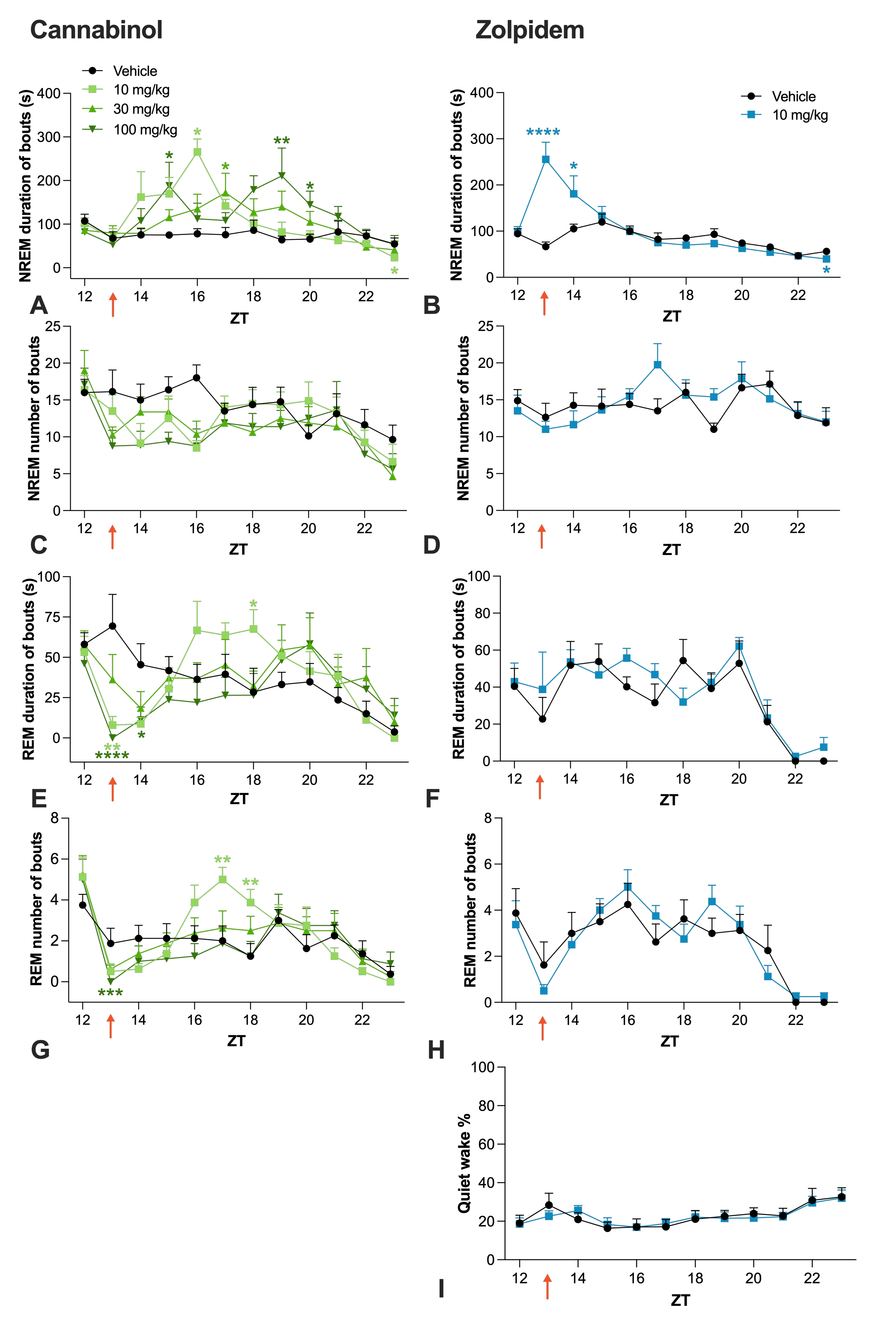
**

**Fig. S1. Effects of CBN and zolpidem on duration and number of NREM and REM bouts in rats. A** CBN and **B** zolpidem increased the mean duration of NREM bouts per hour (treatment and time interactions, χ^2^_30_ = 48.33, *P* = 0.018; χ^2^_10_ = 96.691, *P* < 0.0001 respectively; zolpidem main effect of treatment, χ^2^_1_ = 78.195, *P* < 0.0001). **C** CBN but not **D** zolpidem decreased the number of NREM bouts per hour (main effect of CBN treatment (χ^2^_3_ = 9.382, *P* = 0.025). **E** CBN initially decreased REM bout duration and then increased duration of REM bouts (main effect CBN, χ^2^_3_ = 25.253, *P* < 0.0001; CBN by time interaction, χ^2^_30_ = 62.039, *P* = 0.0005). **F** Zolpidem did not affect duration of REM bouts. **G** CBN biphasically influenced REM bout number (CBN main effect; χ^2^_3_ = 10.471, *P* = 0.015, CBN by time interaction χ^2^_30_ = 56.926, *P* = 0.002). **H** Zolpidem did not affect number of REM bouts nor **I** % quiet wake. Time is expressed relative to lights on (ZT). Dunn–Šidák corrected multiple comparisons test **P <* 0.05, ** *P <* 0.01, ****P <* 0.001, **** *P <*0.0001. Error bars display +/- SEM, n = 8 per group. NREM = non-rapid eye movement sleep; REM = rapid eye movement sleep; ZT = zeitgeber time.


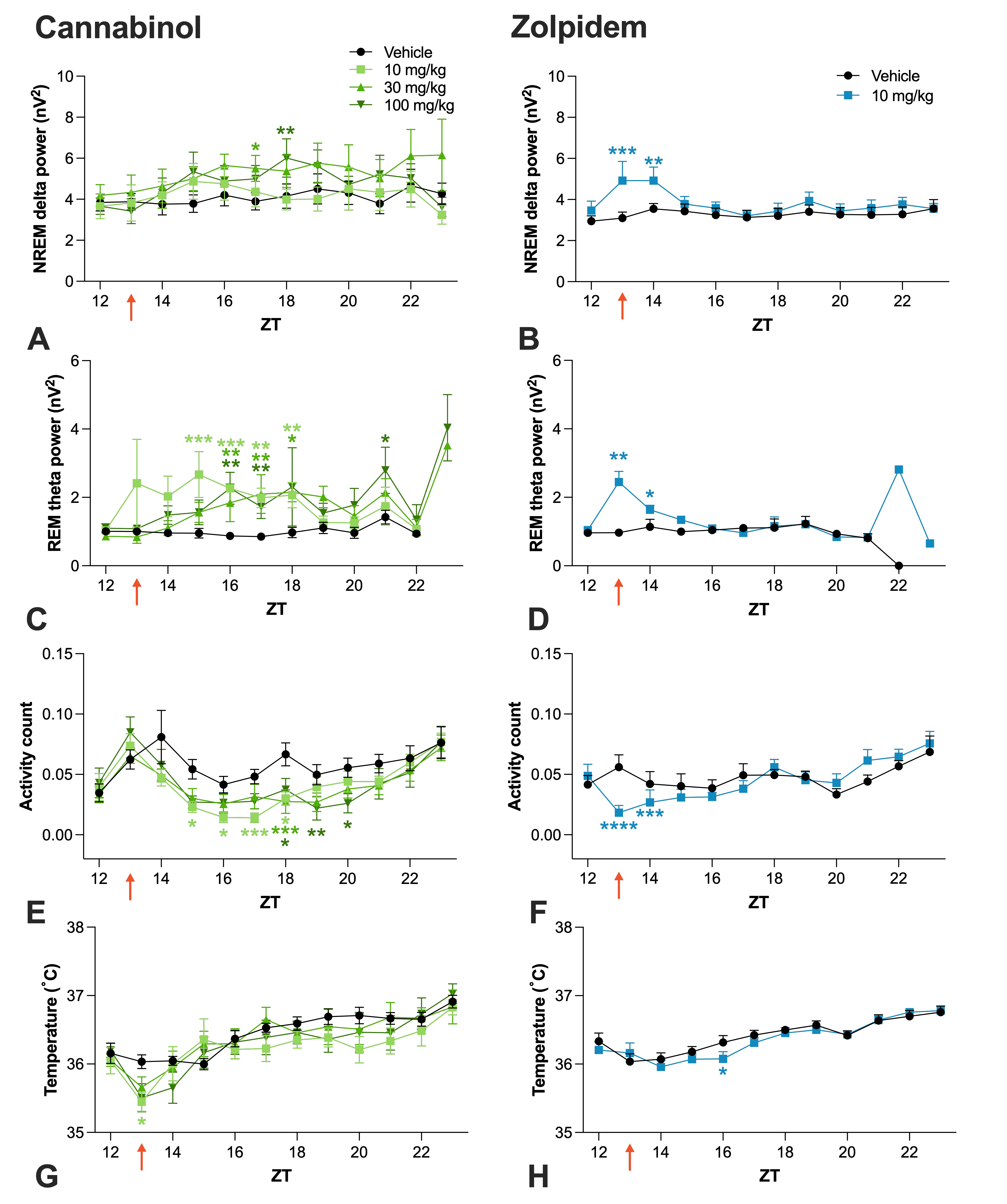


**Fig. S2. Effects of CBN and zolpidem on EEG power spectra, locomotor activity and body temperature. A** CBN nor **B** zolpidem increased NREM delta power (no treatment or treatment by time interactions), although there were increases at specific time-points for both compounds. **C** CBN and **D** zolpidem increased REM theta power (CBN by time interaction, χ^2^_3_ = 58.439, *P* = 0.002; zolpidem main effect, χ^2^_3_ = 17.222, *P* = 0.045). **E** CBN and **F** zolpidem decreased locomotor motor activity counts (CBN by time interaction, χ^2^_30_ = 49.620, *P* = 0.032; zolpidem by time interaction, χ^2^_30_ = 35.940, *P* = 0.0002). **G** CBN and **H** zolpidem did not robustly effect body temperature (no treatment or treatment by time interactions). Time is expressed relative to lights on (ZT). Dunn–Šidák corrected multiple comparisons test **P <* 0.05, ** *P <* 0.01, ****P <* 0.001, **** *P <*0.0001. Error bars display +/- SEM, n = 8 per group. NREM = non-rapid eye movement sleep; REM = rapid eye movement sleep; ZT = zeitgeber time.


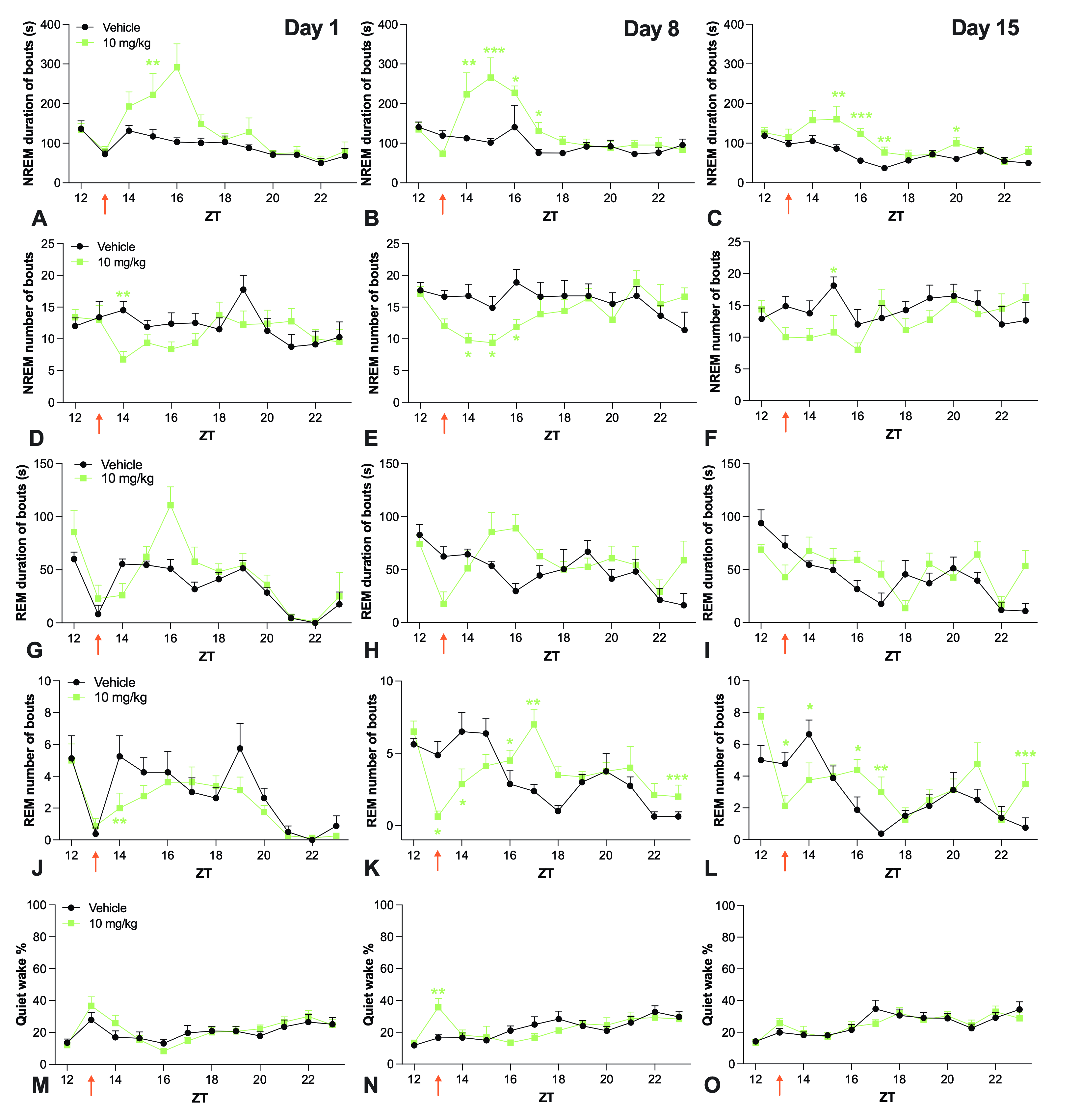


**Fig. S3. Effects of repeated CBN exposure on duration and number of NREM and REM sleep bouts in rats.** CBN increased NREM bout duration over the days 1 **A**, 8 **B** and 15 **C** (CBN and time interaction, χ^2^_10_ = 69.035, *P* < 0.0001), which was not subject to tolerance (no CBN, time and day interaction, *P* > 0.05). CBN decreased NREM bout number over the days 1 **D**, 8 **E** and 15 **F** (CBN and time interaction, χ^2^_10_ = 39.798, *P* < 0.0001), which was not subject to tolerance (no CBN, time and day interaction, *P* > 0.05). CBN affected REM bout duration over the days 1 **G**, 8 **H** and 15 **I** (CBN and day interaction, χ^2^_2_ = 6.585, *P* = 0.037). CBN affected REM bout number over the days 1 **J**, 8 **K** and 15 **L** (CBN main effect, χ^2^_1_ = 10.990, *P* = 0.001; CBN and time interaction, χ^2^_10_ = 83.939, P < 0.0001), which differed across days (CBN, time and day interaction, χ^2^_20_ = 52.542, *P* < 0.0001). CBN affected % quiet wake over days 1 **M**, 8 **N** and 15 **O** (CBN main effect, χ^2^_1_ = 5.367, *P* = 0.021; CBN and time interaction, χ^2^_10_ = 25.817, *P* = 0.004), which was not subject to tolerance (no CBN, time or day interaction, *P* > 0.05). Time is expressed relative to lights on (ZT). Dunn–Šidák corrected multiple comparisons test **P <* 0.05, ** *P <* 0.01, ****P <* 0.001, **** *P <*0.0001. Error bars display +/- SEM, n = 8 per group. NREM = non-rapid eye movement sleep; REM = rapid eye movement sleep; ZT = zeitgeber time.

**
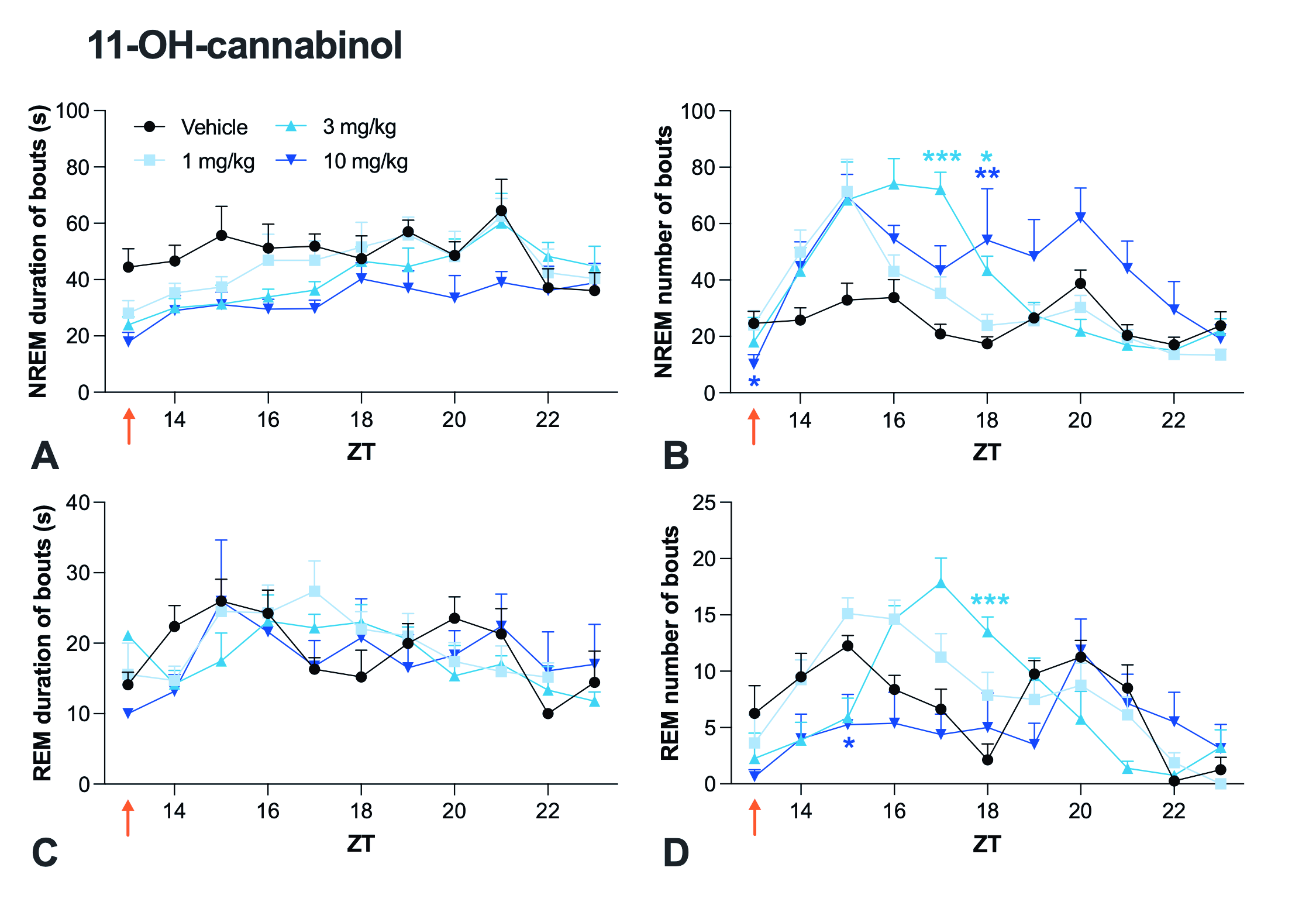
**

**Fig. S4. Effects of 11-OH-CBN on duration and number of NREM and REM sleep bouts in rats.** 11-OH-CBN significantly decreased **A** duration of NREM bouts (11-OH-CBN main effect, χ^2^_3_ = 17.231, *P* = 0.0006) and increased **B** NREM bout number (11-OH-CBN main effect, χ^2^_3_ = 12.712, *P* = 0.005; 11-OH-CBN and time interaction, χ^2^_30_ = 104.226, *P* < 0.0001). 11-OH-CBN did not affect duration of **C** REM bouts, however affected **D** REM bout number (11-OH-CBN main effect, χ^2^_3_ = 9.792, *P* = 0.02; 11-OH-CBN and time interaction, χ^2^_30_ = 127.103, *P* < 0.0001). Time is expressed relative to lights on (ZT). Dunn–Šidák corrected multiple comparisons test **P <* 0.05, ** *P <* 0.01, ****P <* 0.001, **** *P <*0.0001. Error bars display +/- SEM, n = 8 per group. NREM = non-rapid eye movement sleep; REM = rapid eye movement sleep; ZT = zeitgeber time.


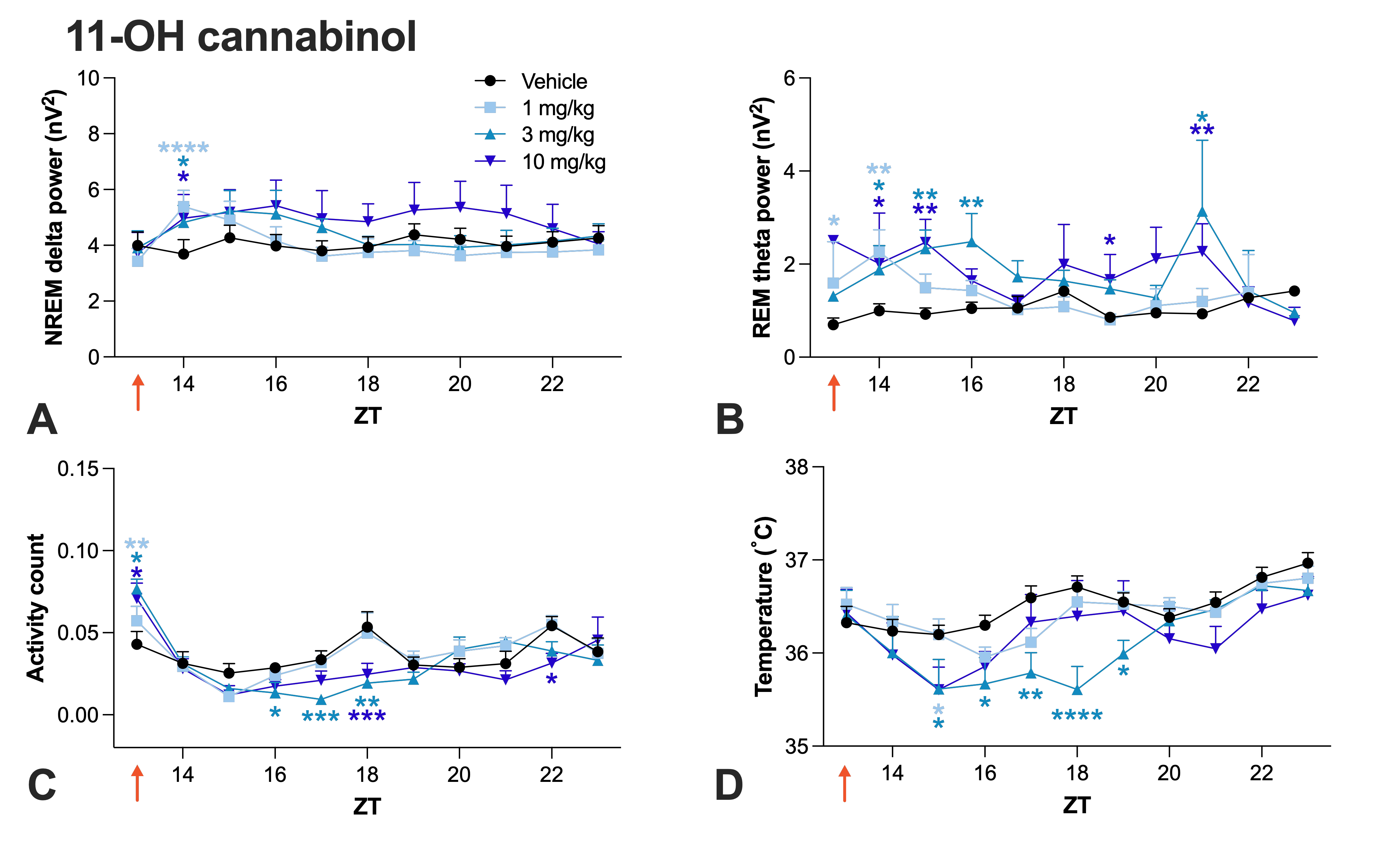


**Fig. S5. Effects of 11-OH-CBN on EEG power spectra, locomotor activity and body temperature.** 11-OH-CBN significantly increased **A** NREM delta power (11-OH-CBN by treatment interaction, χ^2^_3_ = 50.500, *P* = 0.011) and **B** REM theta power (11-OH-CBN main effect, χ^2^_3_ = 38.631, *P* = 0.025). **C** 11-OH-CBN had a biphasic effect on locomotor activity (11-OH-CBN main effect, χ^2^_3_ = 11.758, *P* = 0.008; 11-OH-CBN and time interaction, χ^2^_30_ = 81.362, *P* < 0.00001). **D** 11-OH-CBN decreased body temperature (11-OH-CBN by time interaction, χ^2^_30_ = 44.811, *P* = 0.040). Time is expressed relative to lights on (ZT). Dunn–Šidák corrected multiple comparisons test **P <* 0.05, ** *P <* 0.01, ****P <* 0.001, **** *P <*0.0001. Error bars display +/- SEM, n = 8 per group. NREM = non-rapid eye movement sleep; REM = rapid eye movement sleep; ZT = zeitgeber time.

**Table S1.** Pharmacokinetics of CBN and metabolites 11-OH-CBN and 11-COOH-CBN in rat plasma and brain samples following administration of CBN 10 mg/kg IP. n = 4 rats per group.

|  | **CBN** | **11-OH-CBN** | **11-COOH-CBN** |
| --- | --- | --- | --- |
| **Plasma** |  |  |  |
| C_max_ (ng/mL) | 374 ± 34 | 170 ± 18 | 169 ± 38 |
| t_max_ (hour) | 0.5 | 1 | 1 |
| t_½_ (hours) | 1.93 | 2.18 | 4.52 |
| AUC_∞_ (ng hour/mL) | 775 | 493 | 1181 |
| **Brain** |  |  |  |
| C_max_ (pg/mg brain) | 373 ± 26 | 419 ± 37 | 28 ± 5 |
| t_max_ (hour) | 2 | 2 | 2 |
| t_½_ (hours) | 2.20 | 2.08 | 3.48 |
| AUC_∞_ (pg hour/mg brain) | 1623 | 1539 | 137 |
| Brain/plasma ratio | 2.09 | 3.12 | 0.12 |

**Fig. S6. In-house HPLC analysis or CBN showing 100% purity.**

**References**

1 Fernandes M, Schabarek A, Coper H, Hill R. Modification of delta9-THC-actions by cannabinol and cannabidiol in the rat. Psychopharmacologia. 1974;38(4):329-38.

2 Jarbe TU, Hiltunen AJ. Cannabimimetic activity of cannabinol in rats and pigeons. Neuropharmacology. 1987;26(2-3):219-28.

3 Neckelmann D, Ursin R. Sleep stages and EEG power spectrum in relation to acoustical stimulus arousal threshold in the rat. Sleep. 1993;16(5):467-77.

4 Kevin RC, Vogel R, Doohan P, Berger M, Amminger GP, McGregor IS. A validated method for the simultaneous quantification of cannabidiol, Delta(9) -tetrahydrocannabinol, and their metabolites in human plasma and application to plasma samples from an oral cannabidiol open-label trial. Drug Test Anal. 2021;13(3):614-27.

5 Hawkins NA, Anderson LL, Gertler TS, Laux L, George AL, Jr., Kearney JA. Screening of conventional anticonvulsants in a genetic mouse model of epilepsy. Ann Clin Transl Neurol. 2017;4(5):326-39.

6 Sadamitsu Y, Okumura A, Saito K, Yamada T. Kolbe-Schmitt type reaction under ambient conditions mediated by an organic base. Chem Commun (Camb). 2019;55(66):9837-40.

7 Abe H, Horii A, Matsumoto S, Shiro M, Inouye M. D3h-symmetrical hydrogen-bonding unit as a saccharide recognition and self-assembling module. Org Lett. 2008;10(13):2685-8.

8 Killander D, Sterner O. Synthesis of the Bioactive Benzochromenes Pulchrol and Pulchral, Metabolites of Bourreria pulchra. European Journal of Organic Chemistry. 2014;2014(8):1594-96.

9 Novak J, Salemink CA. Cannabis. Part 27. Synthesis of 8-, 10-, and 11-oxygenated cannabinols. J Chem Soc, Perkin Trans 1. 1983:2867-71.

10 Bates D, Mächler M, Bolker B, Walker S. Fitting linear lixed-effects models using lme4. Journal of Statistical Software. 2015;67(1):1-48.

11 Brooks ME, Kristensen K, van Benthem KJ, Magnusson A, Berg CW, Nielsen A, et al. glmmTMB balances speed and flexibility among packages for zero-inflated generalized linear mixed modeling. The R journal. 2017;9(2):378-400.

12 R Core Team. R: A language and environment for statistical computing. R Foundation for Statistical Computing. 2022. <https://www.R-project.org/>.

13 Fox J, Weisberg S. An R companion to applied regression*.* 3^rd^ ed. Sage Publications: Thousand Oaks, California; 2018.

14 Zuur A, Ieno EN, Walker N, Saveliev AA, Smith GM. Mixed effects models and extensions in ecology with R*.* Springer: New York; 2009.
